# A set of monomeric near-infrared fluorescent proteins for multicolor imaging across scales

**DOI:** 10.1038/s41467-019-13897-6

**Published:** 2020-01-13

**Authors:** Mikhail E. Matlashov, Daria M. Shcherbakova, Jonatan Alvelid, Mikhail Baloban, Francesca Pennacchietti, Anton A. Shemetov, Ilaria Testa, Vladislav V. Verkhusha

**Affiliations:** 1 Department of Anatomy and Structural Biology and Gruss-Lipper Biophotonics Center, Albert Einstein College of Medicine, Bronx, New York, 10461 USA; 2 Department of Applied Physics and Science for Life Laboratory, KTH Royal Institute of Technology, Stockholm, Sweden; 3 Medicum, Faculty of Medicine, University of Helsinki, 00029 Helsinki, Finland

**Keywords:** Biological techniques, Fluorescence imaging, Protein design, Super-resolution microscopy

## Abstract

Bright monomeric near-infrared (NIR) fluorescent proteins (FPs) are in high demand as protein tags for multicolor microscopy and in vivo imaging. Here we apply rational design to engineer a complete set of monomeric NIR FPs, which are the brightest genetically encoded NIR probes. We demonstrate that the enhanced miRFP series of NIR FPs, which combine high effective brightness in mammalian cells and monomeric state, perform well in both nanometer-scale imaging with diffraction unlimited stimulated emission depletion (STED) microscopy and centimeter-scale imaging in mice. In STED we achieve ~40 nm resolution in live cells. In living mice we detect ~10^5^ fluorescent cells in deep tissues. Using spectrally distinct monomeric NIR FP variants, we perform two-color live-cell STED microscopy and two-color imaging in vivo. Having emission peaks from 670 nm to 720 nm, the next generation of miRFPs should become versatile NIR probes for multiplexed imaging across spatial scales in different modalities.

## Abstract (web summary)

Monomeric near-infrared (NIR) fluorescent proteins (FPs) from bacterial phytochromes bring potential advantages, but their brightness in cells is lower than dimeric NIR FPs. Here the authors develop enhanced monomeric NIR FPs enabling imaging across different scales without the trade-off between brightness and monomeric state.

## Introduction

Imaging in the near-infrared (NIR) part of the spectrum with NIR fluorescent proteins (FPs) has several advantages over imaging with traditional visible FPs. In a NIR (~650–900 nm) optical window, the tissue is more transparent to light due to less absorbance by body pigments and less scattering^1^. This spectral region is also characterized by low autofluorescence and minimal phototoxicity for living cells. Thus, NIR FPs have been used for quantitative imaging in deep tissues in vivo^2^. Also, a use of distant NIR spectral region for imaging allows combination of NIR FPs in multiplexed experiments with visible FP tags, biosensors and optogenetic tools controlled by 450–600 nm light without cross-activation^3^. Thus, NIR FPs are extremely useful for imaging at various scales, from individual molecules to whole organisms; however, their potential has not yet been fully explored due to the lack of NIR FPs that combine all beneficial characteristics, such as high molecular and effective brightness (also called cellular brightness), monomeric state, and being spectrally shifted in relation to each other.

NIR FPs were developed from bacterial phytochromes (BphP)^2,4^. These proteins, similar to their precursor BphP photoreceptors, use a linear tetrapyrrole biliverdin IXα (BV) as a chromophore. BV is the most red-shifted natural chromophore^5,6^. Fortunately, BV is produced in mammalian cells as an intermediate of heme degradation^7^. The majority of NIR FPs are composed of two domains, the PAS and the GAF, minimally required for BV chromophore binding in BphPs^8^. BV sits in the pocket in the GAF domain, whereas the PAS domain covalently binds BV via Cys residue and is needed for stabilization of the whole structure^9,10^. In the blue-shifted NIR FPs, BV was found being covalently bound to Cys in the GAF domain^11,12^. Interestingly, the PAS and the GAF domains form a topological knot structure with the N-terminus being threaded through the loop in the GAF domain^8,13^. Although a single-domain NIR FP based on the GAF domain of cyanobacteriochrome (CBCR) was developed, the current probe named miRFP670nano yields to two-domain BphP-based NIR FPs in molecular brightness^14^.

To date, several series of NIR FPs have been published^15–22^. For BphP-based NIR FPs, the apparent brightness of a FP in mammalian cells is one of the most important properties to be considered^23^. Because apoprotein NIR FPs incorporate BV with different affinities and specificities, and their expression levels and protein stabilities vary, their effective brightness varies too and does not correlate with the molecular brightness. So far NIR FPs of a near-infraRed Fluorescent Protein (iRFP) series demonstrated the highest effective brightness compared to other reported NIR FPs^2^. The iRFPs were developed in five spectral variants, from blue-shifted iRFP670 to red-shifted iRFP720 (the numbers correspond to emission maxima)^15^. That allowed use of iRFPs in multicolor NIR imaging and gave researchers an option to select a NIR FP that best suits their experiment and imaging setup.

Similarly to their precursor canonical BphPs^24^, many NIR FPs are dimers. These include NIR FPs of the iRFP series, which we developed from *Rhodopseudomonas palustris* RpBphP2 and RpBphP6, and FPs of an Infrared Fluorescent Protein (IFP) series developed from *Deinococcus radiodurans* DrBphP. Although IFP1.4 (ref. ^17^) and IFP2.0 (refs. ^18,25^) were originally reported as monomers, later they were found to form dimers and aggregates when used in protein fusions^2,19^.

The dimerization interface in NIR FP dimers is located in the C-terminal α-helixes of the GAF domain^20^. Therefore, monomerization of NIR FPs, mostly obtained from canonical dimeric BphPs, was performed by disruption of the dimeric interface by changing respective hydrophobic amino acid residues in the C-terminus to charged ones. The problem with this approach is that the resulting monomerized NIR FPs, such as Wi-Phy^20^, IFP1.4^17^, and IFP2.0^18^, had relatively poor effective brightness in mammalian cells and/or still formed dimers, limiting their use as protein tags^2^. A use of precursor non-canonical BphPs, such as *Bradyrhizobium* phytochrome (BrBphP) and RpBphP1, which do not dimerize through the C-terminal α-helixes in the GAF domain and naturally contain charged residues in the C-terminus, allowed to engineer the first and until recently the only available monomeric NIR FPs, such as mIFP^19^ and three miRFPs^16^, which fully rely on endogenous BV to fluoresce in mammalian cells.

mIFP and miRFPs were used in various protein fusions, including cytoskeletal filaments, and simultaneously imaged with FPs of green fluorescent protein (GFP) family. However, mIFPs suffer from low effective brightness in mammalian cells and poor photostability. miRFPs were shown to be several-fold brighter^16^. These FPs were used for development of NIR reporters and biosensors, including miSplit for fluorescence complementation assay of protein–protein interaction and RNA imaging, NIR reporter for NF-κB signaling, NIR cell cycle reporters and NIR Rac1 FRET biosensor^16,26^. While miRFPs have highest molecular and effective brightness among monomeric NIR FPs, they yield to dimeric iRFPs having 1.5–3-fold lower effective brightness in mammalian cells. The only monomeric NIR FP that reached the effective brightness of dimeric iRFPs is recently reported miRFP720 (ref. ^26^). However, other spectral variants of bright monomeric NIR FPs are needed because the excitation maximum of miRFP720 at 702 nm is suboptimal, being away from common 633–640 nm lasers. Moreover, availability of other spectral variants should enable multicolor imaging in NIR.

Here we aim to develop a set of monomeric NIR FPs suitable for imaging across spatial scales and that allow researchers to avoid tradeoffs between high effective brightness and monomeric state. We achieve monomerization of iRFPs without a loss in effective brightness. We also further improve available miRFPs by enhancing their cellular protein stability. We show that the engineered monomeric NIR FPs perform well as protein tags and can be efficiently used in super-resolution stimulated emission depletion (STED) microscopy. We then demonstrate that the same FPs, due to their exceptional effective brightness and NIR spectra, can be applied for quantitative whole-body imaging in living mice. Lastly, the availability of several spectral variants allows multicolor NIR imaging in STED and in vivo.

## Results

### Monomerization of dimeric iRFPs

**Fig. 1:**
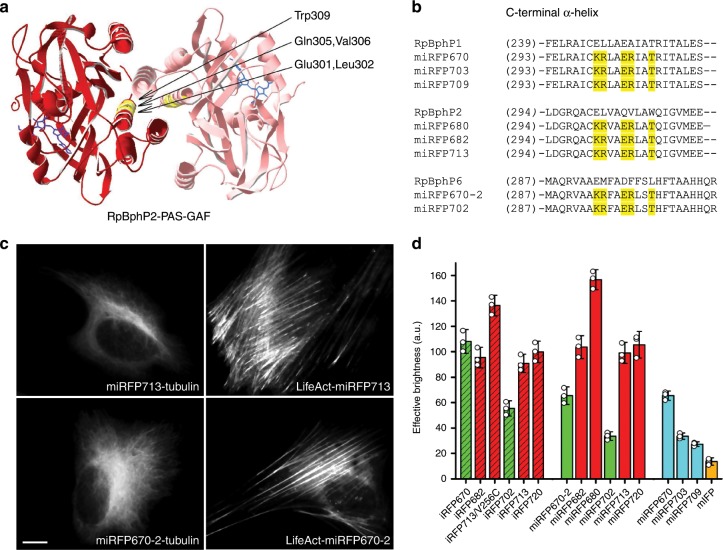
Monomerization of dimeric miRFPs. **a** Dimerization interface in RpBphP2-derived iRFPs. The residues substituted based on alignment with RpBphP1-derived miRFPs are in yellow. The BV chromophore is in blue. The model is based on RpBphP2-PAS-GAF structure (PDB ID: 4R6L). **b** Alignment of C-termini from parental BphPs, i.e. RpBphP1, RpBphP2 and RpBphP6, monomeric RpBphP1-derived miRFPs, and monomerized iRFPs of RpBphP2 and RpBphP6 origins. The substituted residues are marked in yellow. **c** Fusions of miRFP713 (representative monomerized iRFP derived from RpBphP2) and miRFP670–2 (representative monomerized iRFP derived from RpBphP6) labeling actin (LifeAct) and tubulin in live HeLa cells. Scale bar, 10 µm. **d** Brightness of monomerized iRFPs in comparison with dimeric iRFPs and previously reported monomeric NIR FPs. Columns are highlighted according to the origin of NIR FPs (RpBphP2-derived FPs in red, RpBphP6-derived in green and RpBphP1-derived in cyan) and their oligomeric state (dimeric are dashed, monomeric are plain). Error bars are double s.e.m. (*n* = 3; transfection experiments).

We hypothesized that a homology between BphP-based FPs should allow us to monomerize dimeric NIR FPs by transferring charged residues, potentially responsible for disruption of dimeric interface between NIR FPs engineered from different natural BphPs (Supplementary Fig. 1). The similar approach was shown to be successful for engineering of miRFP720 (ref. ^26^). Therefore, to monomerize iRFPs obtained from RpBphP2 and RpBphP6, we made an alignment of the PAS–GAF domains of these BphPs with RpBphP1-derived miRFPs and introduced the amino acid residues 300K/301R/304E/305R/308T from miRFPs into the respective positions in iRFPs (Fig. 1a, b). To obtain spectrally distinct FPs, we monomerized in this way RpBphP2-based iRFP713, iRFP682 and iRFP713/V256C, and RpBphP6-derived iRFP670 and iRFP702. Compared to iRFP682 (ex. 663 nm, em. 682 nm), iRFP713/V256C was previously found to be spectrally similar (ex. 662 nm, em. 680 nm) but having 1.5-fold higher effective brightness^27^.

We tested performance of the monomerized FPs in protein fusions. Since the C-termini are the same in FPs from each of the RpBphP families, we tested one representative FP from each group, RpBphP2-derived miRFP713 and RpBphP6-derived miRFP670–2, in filamentous cytoskeletal fusions with tubulin (α-tubulin) and LifeAct (Fig. 1c and Supplementary Figs. 2–7). While fusions with original dimeric iRFP713 and iRFP670 proteins showed high percentage of expression artifacts, the fusion constructs with miRFP713 and miRFP670-2 showed correct intracellular localization and no aggregates, as expected from monomeric FP tags. Moreover, we analyzed signal-to-background ratios for widefield images of miRFP713-tubulin fusion and similar fusions of the well-established monomeric proteins EGFP and mCherry and found that they are comparable (Supplementary Fig. 8). Analytical ultracentrifugation has further confirmed the monomeric state of the monomerized iRFPs (Supplementary Fig. 9).

We next tested effective brightness of the monomerized iRFPs, miRFP670-2, miRFP702, miRFP680 (obtained from iRFP713/V256C), miRFP682, miRFP713, and reported before miRFP720 alongside dimeric iRFPs, RpBphP1-derived miRFPs and BrBphP-derived mIFP (Fig. 1d). We found that the RpBphP2-derived miRFPs had similar effective brightness as their dimeric precursors. The RpBphP6-derived miRFPs exhibited ~50% decrease in the effective brightness as compared to the precursors. Therefore, bright monomeric NIR FPs, spectrally analogous to (m)iRFP670 and iRFP702/miRFP703 were still needed.

### Engineering of enhanced miRFPs (emiRFPs)

**Fig. 2:**
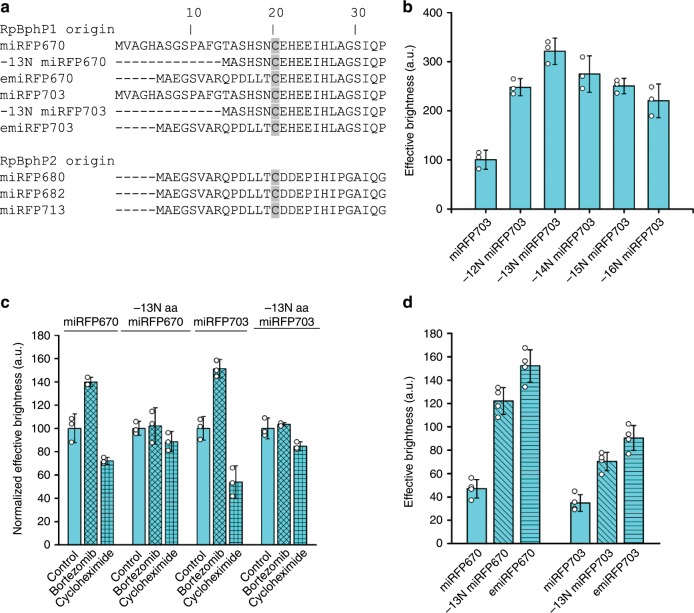
Engineering of enhanced miRFPs. **a** Alignment of N-termini of monomeric NIR FPs derived from RpBphP1 and RpBphP2. **b** Effective brightness of miRFP703 variants with 12–16 amino acid residues removed from the N-terminus. Analysis was performed in COS-1 cells using flow cytometry 72 h after transfection. The effective brightness of the original miRFP670 was assumed to be 100% for normalization. **c** Effective brightness of miRFP670 and miRFP703 and their variants with 13 amino acid residues removed from N-terminus in control cells and after 4 h incubation with 10 µM proteasome inhibitor bortezomib or 50 µg ml^−1^ ribosome blocker cycloheximide in live COS-1 cells. Error bars, double s.e.m. (*n* ≥ 3; transfection experiments). Brightness of FPs in control cells was assumed to be 100% for normalization. **d** Effective brightness of miRFP670 and miRFP703, their variants with 13 amino acid residues removed from N-terminus, and their variants with N-termini of RpBphP2. Measurements were performed in COS-1 cells 72 h after transfection. Effective brightness of miRFP720 was assumed to be 100% for normalization.

To increase the effective brightness of miRFP703, we applied directed molecular evolution consisting of random mutagenesis followed by selection of the brightest clones in bacterial and mammalian cells. We obtained several clones with slight improvement in effective brightness in mammalian cells (less than 50%), which all contained mutations of different amino acid residues in the N-terminus with no obvious pattern. The N-terminus of BphP-based NIR FPs, including miRFPs, is unstructured and cannot be visualized on a protein structure^11,12^. It is not conserved (Fig. 2a and Supplementary Fig. 10a) and can be shortened almost up to the chromophore binding Cys in fusion constructs without substantial loss of fluorescence. However, the N-terminus of the PAS domain is threaded through the figure-of-eight loop in the GAF domain, forming a knot structure^9^. Therefore, it should be important for the folding and stability of the whole protein structure.

We decided to study an effect of N-terminus on effective brightness of miRFP703 systematically. For this, we removed amino acid residues at the N-terminus one by one and compared the brightness of the obtained variants in mammalian cells. Surprisingly, we found that the variant without the first 13 amino acid residues was the brightest showing more than 3-fold increase in effective brightness in HeLa cells, compared to the original miRFP703 (Fig. 2b). Based on alignment (Fig. 2a), we shortened N-termini in miRFPs of RpBphP2 and RpBphP6 origins and mIFP of BrBphP origin, leaving five amino acid residues before the chromophore-binding Cys, as in RpBphP1-derived miRFP703. However, these variants did not demonstrate any improvement in effective brightness (Supplementary Fig. 10). Moreover, we tested and observed similar increase in effective brightness for truncated miRFP670 and miRFP703 and no improvement for truncated NIR FPs of other BphP-origins in different mammalian cell lines, such as HeLa, COS-1 and NIH3T3 (Supplementary Fig. 10). We concluded that it is not the length of the N-terminus but the presence of a specific amino acid sequence in the RpBphP1-derived N-terminus leads to the decrease of effective brightness in cells.

The observed decrease in the effective brightness should result from a decrease in the number of functional FP molecules per cell, because the N-terminus is located outside of the chromophore-binding pocket at the surface and should not influence the molecular brightness or chromophore binding efficiency. We tested how incubation with inhibitors of protein synthesis and degradation affects the effective brightness of miRFP670 and miRFP703 and their truncated versions (Fig. 2c). Inhibition of proteasome by bortezomib resulted in an increase in effective brightness by at least 40% for original miRFPs, whereas almost no change was observed for the truncated versions. Inhibition of the protein synthesis by cycloheximide resulted in the larger decrease in effective brightness of original miRFPs (30%), as compared to the truncated miRFPs (10%). We concluded that the N-terminus of the RpBphP1-based proteins promotes degradation of miRFPs.

We hypothesized that the N-terminus can be transferred from a stable bright NIR FP to the RpBphP1-derived miRFPs to increase their cellular stability in cells and, consequently, effective brightness. We chose to transfer the N-terminus from RpBphP2, since we observed a decrease in the brightness when it was removed from RpBphP2-derived miRFP720 (Supplementary Fig. 10). We replaced the original N-terminus in miRFP670 and miRFP703 with the whole RpBphP2-based N-terminus preceding the chromophore-binding Cys. We compared effective brightness of miRFP670 and miRFP703 having original N-termini, ones shortened by 13 a.a. residues or ones with substituted RpBphP2 N-terminus (Fig. 2d). Seventy-two hours after transfection, the effective brightness of the constructs with N-terminus of miRFP720 was up to 30% higher compared to the shortened constructs. We tested and confirmed the similar increase in the effective brightness for miRFP670 and miRFP703 with the RpBphP2 N-terminus in different cell lines, such as COS-1, HeLa and NIH3T3 (Supplementary Fig. 11). We also exchanged the N-terminus in RpBphP6-derived miRFP670–2 and miRFP702 for the RpBphP2 N-terminus; however, we did not observe an increase in the effective brightness (Supplementary Fig. 12a).

We named the miRFP670 and miRFP703 variants with RpBphP2 N-terminus as emiRFP670 and emiRFP703, respectively, where “e” stands for enhanced. To confirm that emiRFPs exhibit the increased effective brightness due to the increased protein stability, we measured a change in the brightness over time in transiently transfected COS-1 cells (Supplementary Fig. 12b). Ninety-six hours after transfection, the cells expressing original miRFP670 and miRFP703 lost ~40–50% of fluorescence signal as compared to 48 h, whereas the cells expressing emiRFPs lost 10–20% only.

### Properties of the engineered monomeric NIR FPs

Molecular brightness (the product of a molar extinction coefficient and a quantum yield), pH stability and photostability of the monomerized iRFPs were similar to those of their parental iRFPs (Table 1), with an exception of miRFP680 that exhibited twice higher photostability. Apparently, the amino acid substitutions in the dimerization interface had little effect on the chromophore microenvironment in miRFP670–2, miRFP682, miRFP702 and miRFP713. The developed enhanced emiRFP670 and emiRFP703 had these characteristics similar to the parental miRFP670 and miRFP703 too as expected, because the substituted N-terminus is located on the protein surface outside of the chromophore pocket.

**Table 1:** Spectral and biochemical properties of the currently available BphP-derived monomeric NIR FPs.

Monomeric NIR FP	Parental BphP	Ex, nm	Em, nm	Extinction coefficient, M^−1^cm^−1^	Quantum yield, %	Molecular brightness vs. miRFP720, %^a^	Photostability in mammalian cells, *t*_1/2_, s	pKa	Eff. brightness in HeLa cells vs. miRFP720, %^b^	Reference
miRFP670	RpBphP1	642	670	87,400	14.0	204.7	490	4.5	67.9	^16^
miRFP670–2	RpBphP6	643	670	103,000	13.6	234.3	310	4.5	80.8	This paper
emiRFP670*^c^	RpBphP1	642	670	87,400	14.0	204.7	450	4.5	117.0	
miRFP680*	RpBphP2	661	680	94,000	14.5	228.0	980	4.5	156.7	
miRFP682	RpBphP2	663	682	91,000	11.2	170.5	500	4.5	101.8	
miRFP702	RpBphP6	673	702	88,000	8.1	119.2	640	4.5	40.6	
miRFP703	RpBphP1	674	703	90,900	8.6	130.8	650	4.5	49.8	^16^
emiRFP703*	RpBphP1	674	703	90,900	8.6	130.8	700	4.5	77.6	This paper
mIFP	BrBphP	683	705	65,900	6.9	76.1	90	4.5	13.9	^19^
miRFP709	RpBphP1	683	709	78,400	5.4	79.8	500	4.5	29.0	^16^
miRFP713*	RpBphP2	690	713	99,000	7.0	115.9	980	4.5	94.9	This paper
SNIFP	DrBphP	697	720	149,000	2.2	54.8	n.a.	4.5	n.a.	^33^
miRFP720*	RpBphP2	702	720	98,000	6.1	100.0	510	4.5	100	^26^

^a^Molecular brightness is a product of extinction coefficient and fluorescence emission quantum yield

^b^Determined as a mean NIR fluorescence intensity of HeLa cells 48 h after transfection with no supply of exogenous BV

^c^A set of five spectrally distinct monomeric NIR FPs, which is recommended for further use in imaging applications, is marked with asterisks

**Fig. 3:**
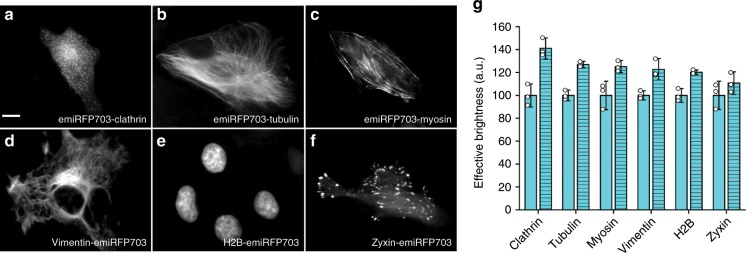
Fusions of emiRFP703 with cellular proteins. **a**–**f** Fusions of emiRFP703 labeling clathrin (**a**), tubulin (**b**), myosin (**c**), vimentin (**d**), H2B (**e**) and zyxin (**f**) in HeLa cells. **g** Effective brightness of miRFP703 (plain) and emiRFP703 (hatch pattern) fusions in HeLa cells 72 h after transfection. Scale bar, 10 µm. Error bars, double s.e.m. (*n* = 3; transfection experiments).

To test the performance of emiRFPs as protein tags in various intracellular structures, we generated a set of constructs including C-terminal fusions of emiRFP703 with tubulin, myosin and clathrin, and N-terminal fusions with H2B, zyxin and vimentin (Fig. 3a–f and Supplementary Figs. 13–15). In all fusion constructs, the tagged proteins demonstrated correct localization in live cells, similar to localization of the similar EGFP and mCherry fusion constructs (Supplementary Figs. 13–15). We next compared the effective brightness of the emiRFP703 fusions with the similar fusions of parental miRFP703 (Fig. 3g). All cells with the emiRFP703 fusion constructs were brighter, having more prominent increase over the parental miRFP703 fusions for the C-terminal emiRFP703 fusions.

### emiRFPs in super-resolution STED microscopy (nanometer scale)

Super-resolution live-cell imaging calls for development of FPs that are sufficiently bright and allow efficient photoswitching or STED, thus requiring less illumination power for imaging. They should also be photostable to withstand multiple cycles of illumination.

Here we show that the emiRFP670 and emiRFP703 can be used for super-resolution imaging of cellular fusions with different proteins such as LAMP1 in lysosomes, H2B in the nucleus, vimentin, clathrin in endosomes, tubulin and myosin (Supplementary Fig. 16). Moreover, the brightness and spectral properties of emiRFP670 and emiRFP703 allow imaging by a standard 640 nm excitation laser that is readily available on most commercial confocal and even super-resolution microscopes. Previously, we demonstrated imaging of miRFP703 in structure-illumination microscopy (SIM) in fixed cells doubling the spatial resolution achievable with conventional techniques^16^.

**Fig. 4:**
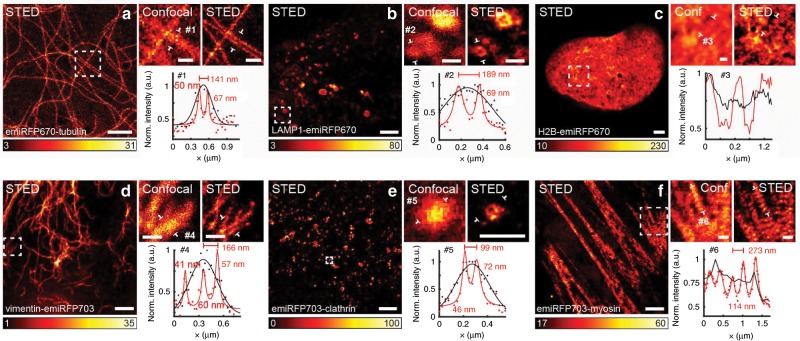
Live-cell STED imaging of emiRFP670 and emiRFP703 fusions. **a** Confocal and STED images of HeLa cells expressing emiRFP670-tubulin. #1 shows a line profile from the zoomed-in region of interest, and shows two tubules 141 nm apart, which cannot be distinguished in the confocal image. **b** Confocal and STED images of U2OS cells expressing LAMP1-emiRFP670. #2 shows a line profile across a lysosome with a diameter of 189 nm and a clearly resolved membrane in the STED data (red solid line). **c** Confocal and STED images of U2OS cells expressing H2B-emiRFP670. #3 is a line profile across a region of the nucleus, which shows several structures not resolvable in the confocal image. **d** Confocal and STED images of HeLa cells expressing vimentin-emiRFP703. #4 shows a line across three filaments in close proximity, which are not resolved in the confocal image. **e** Confocal and STED images of HeLa cells expressing emiRFP703-clathrin. #5 shows a line profile across a small clathrin-coated pit, only visible in the STED data. Line profile is 1 pixel wide. **f** Confocal and STED images of U2OS cells expressing emiRFP703-myosin. #6 shows a line profile across several myosin densities in close proximity, which look homogeneous in the confocal image of the same structure. Line profiles are taken from the raw images, and averaged over a width of 3 pixels unless otherwise stated. A Gaussian was used to fit the confocal data (black solid line) and a Lorentzian to fit the STED data (red solid line). Scale bars are 2 µm for the full images and 500 nm for the inserts showing the zoomed-in regions of interest.

Here, to further overcome the diffraction limit (~230 nm for NIR FPs), and visualize cellular structures at 40–50 nm scale, we use STED microscopy for imaging spectrally distinct emiRFP variants (Fig. 4, Supplementary Fig. 18). The advantage of using STED in the NIR spectral range is the lower-energetic light of such wavelength, allowing focusing of a high power (10–25 mW) STED beam on living cells for achieving the highest spatial resolution. Still, a current challenge in performing STED imaging with previously available NIR probes is to increase the signal and to use even lower STED intensities. The beneficial properties of emiRFPs as bright and photostable NIR tags make them suitable candidates for STED imaging.

We used 640 nm and 775 nm laser light for excitation and STED, respectively. It is important to note that the use of bright NIR emiRFPs and red-shifted lasers allowed imaging of living cells. STED microscopy allowed distinguishing small structures, like filaments in close proximity, clathrin-coated pits as hollow structures and myosin periodic structures, not resolvable in the conventional confocal image. Using the data shown in Fig. 4 and additional analysis on spatial resolution on tubulin filaments (Supplementary Fig. 17), we were able to visualize structures with a full-width half-maximum (FWHM) of 41 nm, and routinely achieving a spatial resolution of 50–70 nm. Importantly, this is comparable to a typical resolution obtained with established STED probes, such as SiR-tubulin dye^28,29^ (Supplementary Fig. 19, Supplementary Table 1 and Supplementary Note 1) and was achieved with half the STED illumination dose.

**Fig. 5:**
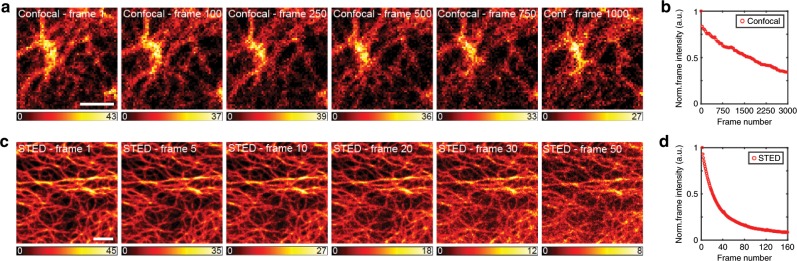
Confocal and STED time-lapse imaging of vimentin-emiRFP703. **a**, **b** A confocal image series (**a**) and **c**, **d** a STED image series (**c**) of live HeLa cells transfected with emiRFP703-vimentin. The graphs (**b**, **d**) show the normalized integrated intensity of the recorded frames (red circles) for both image series, plotted every 40th frame for the confocal for better visualization (**b**) and every frame for STED (**d**). The displayed intensity scale has been adjusted per frame from zero to the maximum in respective frame. Scale bars, 2 µm.

As STED imaging requires photostable FPs, we directly tested how many consecutive frames can be obtained without a substantial loss in brightness (Fig. 5). For confocal microscopy, we could detect 1500 frames before the FP brightness decreased in half. For STED, 20 frames could be obtained before the FP brightness decreased in half, and more than 50 frames could be imaged without a loss in image quality. Thus, emiRFPs perform well in comparison with previously reported far-red FPs in terms of photostability and allow use of substantially lower STED illumination dose (11–36-fold lower than for other far-red FPs) (Supplementary Table 2 and Supplementary Note 1).

**Fig. 6:**
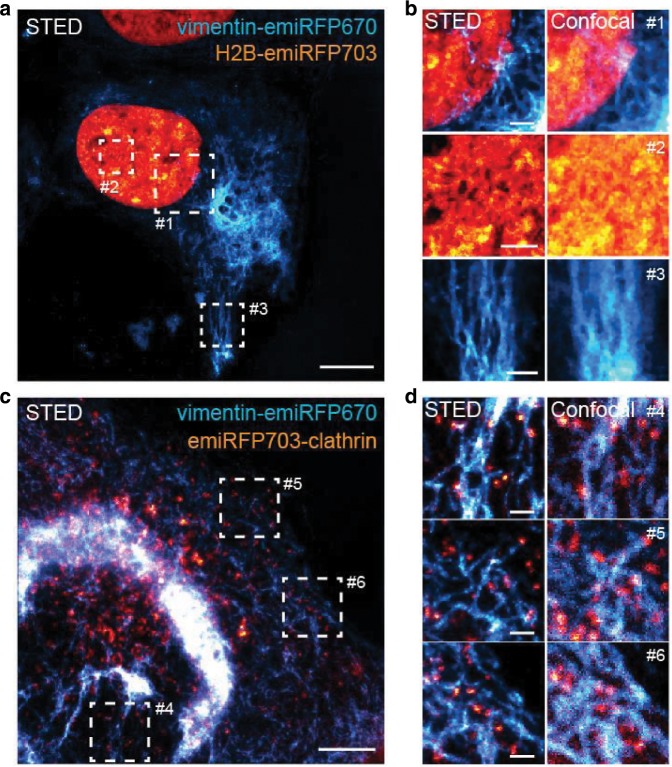
Two-color STED imaging of emiRFP670 and miRFP703 fusions. **a** STED image of a live U2OS cell expressing vimentin-emiRFP670 (cyan) and H2B-emiRFP703 (orange). Scale bar, 10 µm. **b** Three zoom-ins of the regions of interest #1–3 from image **a**. Scale bars, 2 µm. **c** STED image of a live U2OS cell expressing vimentin-emiRFP670 (cyan) and emiRFP703-clathrin (orange). Scale bar, 5 µm. **d** Three zoom-ins of the regions of interest #4–6 from image **c**. Scale bars, 1 µm.

Having spectrally distinct emiRFPs further opens a possibility for a multiplexed imaging. We imaged co-expressed vimentin-emiRFP670 and H2B-emiRFP703 or emiRFP703-clathrin by two-color STED in living cells (Fig. 6). STED imaging was performed with two excitation lasers, 585/610 nm for emiRFP670 and 670 nm for emiRFP703, and two emission detection channels, 625–660 nm for emiRFP670 and 710–755 nm to collect emiRFP703 emission. The images were recorded in a line-by-line alternating fashion for the two spectrally separated channels. The fluorescent signal of each emiRFP was either directly spectrally separated in the imaging or extracted by using spectral unmixing since the emission spectra of emiRFP670 slightly overlaps with that of emiRFP703. Thus, we demonstrate the possibility of a two-color STED imaging in the NIR spectral range with two FPs.

### emiRFPs in whole-body imaging in living mice (centimeter scale)

**Fig. 7:**
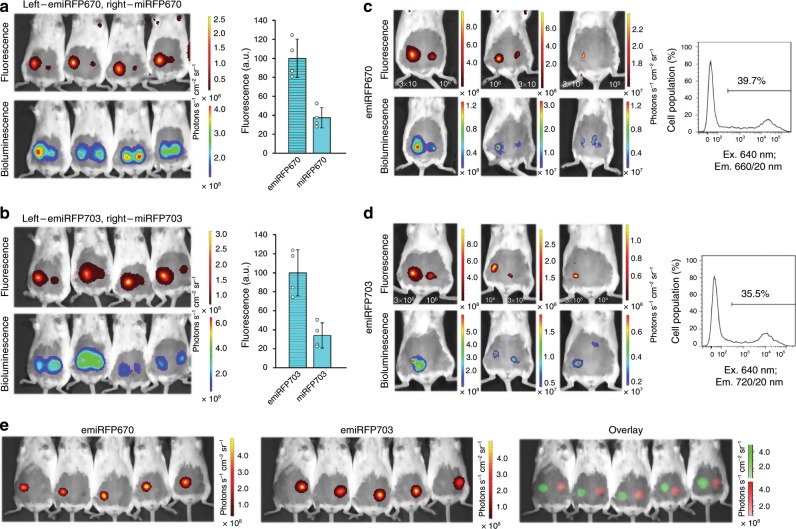
Performance of emiRFPs in vivo. **a**, **b** Comparison of emiRFP670 with parental miRFP670 (**a**) and emiRFP703 with parental miRFP703 (**b**). Fluorescence (top row) and bioluminescence (bottom row) images of living mice injected with 3 × 10^6^ COS-1 cells expressing emiRFP670 or emiRFP703 (left) and miRFP670 or miRFP703 (right). Cells were co-transfected with Rluc8. The filter sets were 640/20 nm excitation and 680/30 nm emission (**a**) or 675/20 nm excitation and 720/30 nm emission (**b**). Bar plots at the right show quantified mean fluorescence intensities (normalized to bioluminescence) that correspond to **a** and **b**. Error bars, double s.e.m. (*n* = 3 experiments). **c**, **d** Minimal number of detectable fluorescent cells. Fluorescence (top row) and bioluminescence (bottom row) images of living mice injected with various quantities of COS-1 cells expressing emiRFP670 (**c**) or emiRFP703 (**d**) and co-transfected with Rluc8. The filter sets were 640/20 nm excitation and 680/30 nm emission (**c**) or 675/20 nm excitation and 720/30 nm emission (**d**). Transfection efficiency of injected COS-1 cells obtained by FACS analysis shown on the right of each panel. **e** Two-color imaging of emiRFP670 and emiRFP703. Fluorescence images of living mice injected with 3 × 10^6^ COS-1 cells expressing emiRFP670 (left row) and emiRFP703 (middle row) and its unmixed overlay (bottom row) are shown. Images were acquired in 19 spectral channels using IVIS Spectrum instrument and spectrally unmixed.

We next tested the performance of emiRFPs as markers for non-invasive whole-body imaging. First we compared the brightness of emiRFPs with parental miRFPs in vivo. For this, we implanted the same amounts of COS-1 cells expressing the respective NIR FPs in mammary glands of mice. The NIR fluorescence signals were normalized to bioluminescence of co-expressed Rluc8 to account for transfection efficiency and cell quantity. The brightness of the emiRFP-expressing cells in vivo was ~2.8-fold higher than that of the respective parental miRFP cells (Fig. 7a, b), consisting with the COS-1 imaging in cell culture (Fig. 2d).

We then assessed the minimal detectable number of the implanted cells expressing emiRFPs. For this, we injected in mammary glands various amounts of transfected cells and found that we were able to detect 1.2 × 10^5^ and 1.0 × 10^5^ fluorescent cells for emiRFP670 and emiRFP703, respectively (Fig. 7c, d).

We next explored the spectral diversity of emiRFP670 and emiRFP703 and tested them for simultaneous imaging of two cellular populations in vivo. Cells transfected with either emiRFP670 or emiRFP703 were well spectrally resolved in living mice by applying the standard spectral unmixing algorithm in an IVIS Spectrum imaging system (Fig. 7e).

## Discussion

**Fig. 8:**
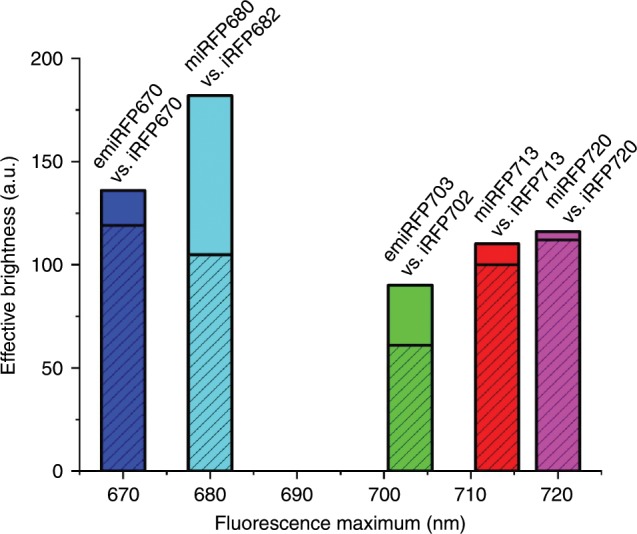
Brightness of the monomeric miRFPs compared to that of spectrally similar dimeric iRFPs. The effective brightness of (e)miRFPs (plain) and the respective spectral variants of dimeric iRFPs (dashed) is shown. The heights of the columns correspond to the mean effective brightness obtained in HeLa cells 48 h after transfection, normalized to the brightness of iRFP713. The columns are plotted on the x axis in accordance with the wavelength of the emission peak of the corresponding NIR FP.

We have developed several spectrally distinct bright monomeric NIR FPs, proposed as the expanded set for various imaging applications, from microscopy to in vivo imaging. This set consists of the brightest FP versions reported in this work, such as emiRFP670, miRFP680, emiRFP703, miRFP713 and reported earlier miRFP720 (ref. ^26^). Under the common standard conditions (HeLa cells, 48 h after transfection), the engineered miRFPs are up to 1.7-fold brighter than dimeric iRFPs^15^ (Fig. 8) and almost 2-fold brighter than previously published miRFPs (Table 1).

For engineering of the miRFPs, we explored the homology between NIR FPs and were able to transfer beneficial sequences in respective protein structure elements between NIR FPs of different BphP origins. We suggest that the method of the NIR FP monomerization by transferring of the specific amino acid residues from the C-terminal α-helix of RpBphP1-derived miRFPs to the C-termini of other NIR FPs performed in this work is universal and can be applied for monomerization of dimeric NIR FPs of any BphP origin with a minimal loss in effective brightness in mammalian cells.

To increase the effective brightness of RpBphP1-derived miRFPs, we exchanged their original N-terminus with the N-terminus from RpBphP2. Our results (Figs. 2, 3 and Supplementary Figs. 10–12) indicate that the RpBphP1-derived N-terminus contains the sequence that causes an increased protein degradation by proteasome. However, computer analysis did not allow us to identify this sequence. There were no lysine residues and, consequently, no potential sumoilation and ubiquitination sites. Clearly, the N-terminus of any BphP-derived NIR FP affects protein stability in cells. That is expected because the N-terminus of the PAS domain is threaded through the loop in the GAF domain forming a knot structure. Moreover, this knot is tightened by a covalent binding of BV located in the GAF domain to the Cys that is located right after the N-terminus in the PAS domain. The importance of the stability of the whole protein structure for denaturation and its correlation with the effective brightness were acknowledged previously^27^.

Enhanced emiRFP670 and emiRFP703 combine monomeric state, exceptional effective brightness, and good photostability, making them suitable fluorescent tags for live-cell STED microscopy. STED imaging requires a relatively high-power STED beam to confine the emission signal to a sub-diffraction-sized volume^30,31^, which can be potentially toxic to cells. STED with far-red, or better, NIR FPs minimizes this risk^32^. Bright monomeric emiRFPs satisfy the requirements of low toxicity to cells and high photostability, enabling NIR STED imaging of live cells.

We demonstrated STED imaging of the emiRFP fusions with a resolution approaching 40 nm (Fig. 4 and Supplementary Fig. 17). The brightness of the optimized emiRFPs allowed to perform NIR STED with the widely used 640 nm excitation laser and without addition of exogenous BV chromophore, in contrast to STED performed earlier with SNIFP protein with low quantum yield (2.2%) of DrBphP origin^33^. The NIR FPs are excited at or close to their peak by 640–670 nm lasers. In contrast, the far-red GFP-like FPs previously used for live-cell STED, such as E2-Crimson^34^, mGarnet^35^, TagRFP657^36^ and mNeptune2^37,38^, have to be excited at the red edge of their spectrum. Moreover, the far-red FPs suffer from lower photostability, allowing to take no more than 20 consecutive frames without loss in image quality^35,39^, as compared to 50 frames obtained with emiRFPs. Furthermore, the distinct excitation and emission spectra of emiRFP670 and emiRFP703 enabled two-color NIR STED with FPs, which before was only possible with organic fluorophores^40^.

In addition to multicolor super-resolution microscopy, the most popular application of NIR FPs is in vivo imaging^2^. We validated the performance of emiRFPs in living mice. The cells expressing emiRFPs produced several-fold brighter fluorescence signal 1–2 mm deep in mouse tissues than the cells with parental miRFPs. The minimal number of detectable cells 10^5^ is comparable or even lower than previously reported detection limits for NIR FPs^14,41^. Spectral difference between emiRFP670 and emiRFP703 allowed their two-color imaging in vivo.

We envision that the proposed set of five spectrally distinct monomeric NIR FPs, consisting of emiRFP670, miRFP680, emiRFP703, miRFP713 and miRFP720 (ref. ^26^), should replace the spectrally similar dimeric iRFPs as the state-of-the-art genetically encoded NIR probes. The enhanced miRFPs should become versatile protein tags and building blocks for biosensors, thus, opening broad possibilities for multicolor NIR imaging at both subcellular and organismal scales.

## Methods

### Design of plasmids

To generate plasmids encoding the monomerized NIR FPs, site-specific mutagenesis was performed with QuikChange mutagenesis kit (Agilent Technologies). Similarly, the constructs encoding the emiRFPs and miRFP variants with shortened N-termini were generated using QuikChange mutagenesis kit. *Escherichia coli* TOP10 bacteria strain (Invitrogen) was used for transformation. The mIFP-N1 (#54620), mCherry-tubulin (#55148), vimentin-EGFP (#56439), vimentin-mCherry-N1 (#55158) and H2B-mCherry (#20972) encoding plasmids were obtained from Addgene.

For protein tagging and labeling of intracellular structures study, the fragments encoding emiRFPs were amplified, digested with AgeI and NotI restriction enzymes and ligated with similarly digested cloning vectors for N-terminal fusions (for H2B, vimentin and zyxin) as previously described^16^. For C-terminal fusions (tubulin, clathrin and myosin), the substitution was introduced with QuikChange mutagenesis kit. See Supplementary Table 3 for sequences of the primers used in this study.

### Protein characterization in vitro

For expression in bacteria, the miRFPs were cloned into pBAD/His-B vector (Life Technologies/Invitrogen). LMG194 or TOP10 host cells (Invitrogen) were used for protein expression. A pWA23h plasmid encoding HO from *Bradyrhizobium* ORS278 (hmuO) under the rhamnose promoter was co-transformed with a pBAD/His-B plasmid encoding a FP. Bacterial cells were incubated overnight at 37 °C in RM minimal medium with ampicillin and kanamycin. To start protein expression, 0.002% arabinose and 0.02% rhamnose were added. After growing for 12 h at 37 °C, the cells were incubated at 18 °C for 24 h. Proteins were purified with Ni-NTA agarose (Qiagen). For elution, PBS containing 100 mM EDTA was used instead of imidazole. Then the samples were desalted using PD-10 desalting columns (GE Healthcare).

For recording of fluorescence and absorbance spectra, FluoroMax-3 spectrofluorometer (Jobin Yvon) and Hitachi U-2000 spectrophotometer were used, respectively. For determination of extinction coefficient, we obtained a ratio between the maximum absorbance of the main peak at Q band and the side peak at Soret band and assumed that extinction coefficient at Soret band corresponds to 39,900 M^−1^ cm^−1^ (refs. ^17,22^). For determination of quantum yield, we measured fluorescence spectra of purified proteins in parallel with an equally absorbing Nile blue dye (quantum yield is 0.27 in an acidic ethanol^25^) and previously developed dimeric iRFPs and compared the signal at several dilutions. pH titrations were done using a series of buffers (100 mM sodium acetate, 300 mM NaCl for pH 2.5–5.0 and 100 mM NaH_2_PO_4_, 300 mM NaCl for pH 4.5–9.0). For determination of photostability, FPs in transiently transfected HeLa cells were imaged at different time periods, as described in the widefield fluorescence microscopy section. Obtained raw data were normalized to corresponding absorbance spectra and extinction coefficients of the proteins, the spectrum of 200 W Me-Ha arc lamp and the transmission of 665/45 nm photobleaching filter.

Analytical ultracentrifugation was conducted at 20 °C and 58,000 r.p.m. with an Optima XL-I centrifuge (Beckman Coulter) using the AN-60Ti rotor and the absorption optics set to 645 nm. Sednterp v.20120828beta software was used to calculate the partial specific volume of the proteins from their sequence and the density and viscosity of the buffers. The sedimentation parameters were corrected to standard conditions (20,w) using these values. For sedimentation velocity (SV) experiments, 350 ml of protein sample and an equal volume of PBS buffer were loaded into two-sector cell assemblies with the protein concentration corresponding to A645 E0.9. Fifty scans were collected over the course of a centrifuge run. A subset of scans, beginning with those where a clear plateau was evident between the meniscus and the boundary, was selected for time-derivative analysis using DCDTþ v.2.4.2 software^42^.

### Cell culture

HeLa CCL-2, HEK293, NIH3T3 and COS-1 cells were obtained from ATCC and grown in high-glucose DMEM medium (HyClone) containing 10% (v/v) FBS (Gemini Bio-Products), 0.5% (v/v) penicillin–streptomycin (Mediatech). All cell lines were grown at 37 °C in a humidified 5% CO_2_ atmosphere. Transfection of plasmids was performed with an Effectene reagent (Qiagen) according to the manufacturer’s protocol. Cells were not tested for mycoplasma contamination or cross-contamination.

### Widefield fluorescence microscopy

Live HeLa, HEK293, NIH3T3 and COS-1 cells were imaged with an Olympus IX81 inverted epifluorescence microscope 72 h after the transfection. The microscope was equipped with a 200-W metal halide arc lamp (Lumen220PRO, Prior), a 60 × 1.35 numerical aperture (NA) oil objective lens (UPlanSApo, Olympus) and an opiMOS sCMOS camera (QImaging). During imaging, cells were incubated in a cell imaging medium (Life Technologies-Invitrogen) at room temperature. The microscope was operated with a SlideBook v.6.0.8 software (Intelligent Imaging Innovations).

### Flow cytometry

Flow cytometry analysis was performed using a BD LSRII flow cytometer. All NIR FPs were excited with a 640-nm solid-state laser. Fluorescence of miRFP670, miRFP670–2, miRFP680 and miRFP682 variants was detected with a 660/20 nm emission filter, and fluorescence of mIFP, miRFP703, miRFP702, miRFP713 and miRFP720 variants was detected with a 730/45 nm emission filter. Minimally 3 × 10^5^ live cells were analyzed in each cell sample. Data were analyzed using a FACSDiva v.8.0.1 and a FlowJo v.7.6.2 software.

### STED microscopy setup

STED imaging has been performed with a custom-built STED set-up. The emiRFP-variants were excited with a 640-nm pulsed diode laser (LDH-D-C-640, PicoQuant) and subsequently depleted with a 775-nm pulsed laser (KATANA 08 HP, OneFive), both operating at 40 MHz. The depletion beam was shaped to a donut in the focal plane by the use of a spatial light modulator (LCOS-SLM X10468–02, Hamamatsu Photonics). The excitation laser and depletion laser beams were coupled together and scanned over the sample using fast galvanometer mirrors (galvanometer mirrors 6215H+ servo driver 71215HHJ 671, Cambridge Technology). The laser beams were focused onto the sample using a HC PL APO 100×/1.40 N.A. Oil STED White objective lens (15506378, Leica Microsystems), through which also the fluorescence signal was collected. After de-scanning and de-coupling of the fluorescence signal, it was put through a confocal pinhole (1.28 Airy disk units) and detected through a bandpass filter (ET705/100m, Chroma Technology) and a notch filter (NF03–785E-25, Semrock) with a free-space APD (SPCM-AQRH-13-TR, Excelitas Technologies).

An infrared laser (CPS980S, Thorlabs), coupled into the objective with a dichroic mirror (T860SPXRXT, Chroma Technology) between the scan and tube lens, is aligned to reflect off the coverslip through total internal reflection. The reflected laser beam is separated from the incoming beam through a D-shaped mirror, and imaged onto a CMOS camera (DMK 33UP1300, The Imaging Source Europe). Through a feedback loop reading the position of the laser beam on the camera chip and subsequently moving a z-piezo (LT-Z-100, Piezoconcept) holding the sample, the focus lock system keeps the same desired focal plane in the sample during acquisitions.

The focus lock and the rest of the microscope is controlled through two separate software; image acquisition and some hardware control is done through the Imspector software (Max-Planck Innovation, Göttingen, Germany) while the rest of the hardware control (SLM, focus lock and 775 nm laser) is done through Python-based custom-written microscope control software Tempesta (https://github.com/TestaLab/Tempesta).

### STED microscopy

Single-color imaging of emiRFP670 and emiRFP703 was done with a 640-nm excitation laser power of 5.6–26.1 µW and a 775-nm depletion laser power of 12–25 mW, both measured at the first conjugate back focal plane of the objective. The pixel size for the STED images was set to 25–30 nm and for the confocal images 25–30 nm or 100 nm. The pixel dwell time for the STED images was 5–50 µs and for the confocal images 10 µs.

Two-color STED imaging of emiRFP670 and emiRFP703 was performed with separation in the excitation and emission. The excitation was done with 585 or 610 nm and 670 nm for the two channels, respectively, while the depletion was done with 775 nm for both channels. The emission was detected in two separate channels following respective excitation, with one channel detecting 625–660 nm and the other detecting 710–755 nm. The 585 or 610 nm excitation laser power setting was 20–60% while the 670 nm excitation laser power setting was 10–50%. The depletion laser power setting was 4–15%. The detection signal was gated and the signal 0.3–6.0 ns after the excitation pulse was recorded. The images were recorded in a line-by-line alternating fashion for the two spectrally separated channels.

All images shown are raw data, except images in Fig. 4e (STED and STED zoom-in) and Fig. 6 (STED and STED zoom-ins) that have been deconvolved with a Richardson-Lucy deconvolution algorithm in Imspector, using a Lorentzian PSF with 30–50 nm FWHM. In order to help visualization, we applied a Gaussian smoothing with 0.6–1 pixel radius (equaling 15–30 nm in STED images) in ImageJ on images in Fig. 4a (STED zoom-in), Fig. 4b (STED zoom-in), Fig. 4c (STED and STED zoom-in), Fig. 4d (STED zoom-in), Fig. 4f (STED and STED zoom-in), Figs. 5c, 6b (confocal zoom-ins) and Supplementary Fig. 16c. Additionally, spectral unmixing was performed on the two-color STED image in Fig. 6a by using the ImageJ Spectral Unmixing plug-in from Joachim Walter (https://imagej.nih.gov/ij/plugins/spectral-unmixing.html).

### Cell culture for STED and confocal imaging

Images in Figs. 4–6 and Supplementary Fig. 16 are of U2OS (ATCC HTB-96) and HeLa (ATCC CCL-2) cells, cultured in Dulbecco’s modified Eagle medium (DMEM) (Thermo Fisher Scientific, 41966029) supplemented with 10% (v/v) fetal bovine serum (Thermo Fisher Scientific, 10270106) and 1% penicillin–streptomycin (Sigma-Aldrich, P4333), and kept at 37 °C and 5% CO_2_ in a humidified incubator. For transfection with the emiRFP-variants, cells were seeded on coverslips in a six-well plate. After 24 h, cells were transfected using Lipofectamine LTX Reagent with PLUS reagent (Thermo Fisher Scientific, 15338100) according to the manufacturer’s instructions. About 24–72 h after transfection, cells were washed in phosphate buffered saline (PBS) solution, placed with phenol-red-free DMEM or Leibovitz’s L-15 Medium (ThermoFisher Scientific, 21083027) in a chamber and imaged at room temperature. In some cases, BV (Sigma-Aldrich, 30891) was added to the cell medium either 1 h (Fig. 6c), 2 h (Fig. 4a, f, Supplementary Fig. 16f) or 24 h (Figs. 5a, c and 6a) before imaging.

### Image quantification and statistical analysis

Data fitting and statistical analysis were performed using an OriginPro v.9.2.196 software (OriginLab). Statistical values including the exact *n* and statistical significance are reported in the respective Figure legends. Line profile fitting with Lorentzian functions for STED images and Gaussian functions for confocal images, both including a background term, were performed using MATLAB (MathWorks).

### Imaging in mice

The Swiss Webster 2- to 3-month-old female mice (National Cancer Institute, NIH) with body weights of 22–25 g were used. To compare brightness of miRFPs with emiRFPs and show possibility of two-color imaging of emiRFPs, COS-1 cells were injected subcutaneously in the interscapular area of Swiss Webster mice. For better imaging, the fur on the bellies of the mice was removed using a depilatory cream. Mice were fed with AIN-93M Maintenance Purified Diet (TestDiet) to reduce the intrinsic autofluorescence level. COS-1 cells were co-transfected with the pmiRFP670-N1, pmiRFP703-N1, pemiRFP670-N1 or pemiRFP703-N1 and pRluc8 plasmids in a 9:1 ratio for comparison study using Effectene (Qiagen). COS-1 cells were transfected with the pemiRFP670-N1 or pemiRFP703-N1 plasmids for two-color study using Effectene (Qiagen). For comparison study, 3 × 10^6^ COS-1 cells in 100 μl of RPMI-1640 media supplemented with 2 mM l-glutamine were injected subcutaneously 72 h after the transfection. For studying of detection limit of emiRFPs, various number of cells in 100 μl of RPMI-1640 media supplemented with 2 mM l-glutamine were injected subcutaneously 72 h after the transfection. For fluorescence and bioluminescence detection, 1 h after the COS-1 cells injection, the animals were imaged using an IVIS Spectrum instrument (PerkinElmer/Caliper Life Sciences). Fluorescence was detected with 640/20 nm excitation and 680/20 nm emission filters for miRFP670 and emiRFP670 or 675/20 nm excitation and 720/30 nm emission filters for miRFP703 and miRFP703. For two-color imaging, spectral unmixing approach was applied. An image was collected on the IVIS Spectrum with 19 filter channels. Bioluminescence was detected with an open emission filter. Throughout the imaging, animals were maintained under anesthesia with 1.5% vaporized isoflurane. Prior to imaging for comparison and imaging limit studies, 80 μg of Inject-A-Lume coelenterazine substrate for Rluc8 (NanoLight Technology) was intravenously injected through a retro-orbital vein. Data were analyzed using Living Image 3.0 software (Perkin Elmer/Caliper Life Sciences).

All animal experiments were performed in an AAALAC-approved facility using protocols approved by the Albert Einstein College of Medicine Animal Usage Committee. Twenty mice were used in this study.

### Reproducibility

The experiments were not randomized. The investigators were not blinded to allocation during the experiments and outcome assessment. No sample-size estimation was performed to ensure adequate power to detect a prespecified effect size.

### Reporting Summary

Further information on research design is available in the Nature Research Reporting Summary linked to this article.

## Supplementary information

Supplementary Information

Reporting Summary

## Data Availability

The main data supporting the findings of this study are available within the Article and its Supplementary materials. The additional data are available from the corresponding author on reasonable request. The emiRFP670, miRFP680, emiRFP703 and miRFP713 nucleotide sequences in GenBank are MN701051, MN701052, MN701053 and MN701054, respectively.
