# Supplementary material for: A set of monomeric near-infrared fluorescent proteins for multicolor imaging across scales
Source: Nat Commun. 2020 Jan 13;11:239. doi: 10.1038/s41467-019-13897-6 (PMC6957686; doi:10.1038/s41467-019-13897-6)
Supplement: Supplementary file 1 — Supplementary Information [file 41467_2019_13897_MOESM1_ESM.pdf]

A set of monomeric near-infrared fluorescent proteins for multicolor imaging across scales

Mikhail E. Matlashov, Daria M. Shcherbakova, Jonatan Alvelid, Mikhail Baloban, Francesca Pennacchietti, Anton A. Shemetov, Ilaria Testa and Vladislav V. Verkhusha

Supplementary Information

Supplementary Table 1. Signal-to-noise ratio (SNR) and signal-to-background ratio (SBR) for emiRFP703 and SiR-tubulin. SNR and SBR were both calculated on the whole image. SNR was calculated as the average signal divided by the standard deviation of the background, without or with the mean background subtracted from the image. SBR was calculated as the average signal divided by the average background.

NIR FP or dye	Structure	SNR (μ_s/σ_b)	SNR (background subtracted) (μ_s/σ_b)	SBR (μ_s/μ_b)	STED power, mW	STED intensity, MW/cm^2	Pixel size, nm	Pixel dwell time, µs	STED dose, kJ/cm^2
emiRFP703	tubulin	5 ± 0.3	3.3 ± 0.2	1.7 ± 0	14-17	5.8-7.1	30	30	49-59
emiRFP670	tubulin	3.9 ± 0.3	3.0 ± 0.3	2.0 ± 0.1	17-25	7.2-10.2	30	30	61-86
emiRFP703	vimentin	5.5 ± 0.4	6.2 ± 0.4	4.5 ± 1.1	12	5.0	30	5	6.7
SiR	tubulin	5 ± 0.3	5.3 ± 0.4	4.7 ± 0.6	30	12.5	30	30	105

Supplementary Table 2. Photostability in STED imaging of emiRFP703 compared to that of the published NIR and far-red FPs. The STED dose is the total energy per area unit delivered to the sample by the STED beam during the acquisition of an image.

NIR FP	Structure	Photostability in mammalian cells, $f_{1/2}$, frames	Photostability in mammalian cells, $f_{1/e}$, frames	STED wavelength, nm	STED power, mW	Pixel size, nm	Pixel dwell time, µs	STED dose, kJ/cm^2	Reference
emiRFP703	vimentin	21	33	775	12	30	5	6.7	this paper
SNIFP	vimentin	8 (from graph)	11 ± 3	860	220	30	10	244	[1]
mGarnet2	vimentin	6 (f.g.)	8 ± 2	775	53	30	30	177	[1]
mGarnet	LifeAct	14 (f.g.)	19 (f.g.)	780	29	40	40	73	[2]
mGarnet	RITA	35 (f.g)	~55 (f.g.)	780	46	40	40	115	[2]
mGarnet2	RITA	~12 (f.g.)	~15 (f.g.)	780	36	40	40	90	[3]
mGarnet	RITA	~13 (f.g.)	~19 (f.g.)	780	36	40	40	90	[3]
mKate2	LifeAct	<2	-	732	20	-	-	-	[4]
mPlum	LifeAct	>2	-	732	20	-	-	-	[4]
TagRFP657	LifeAct	>2	-	732	20	-	-	-	[4]
mNeptune	LifeAct	<8	-	732	47.5	-	-	-	[4]
mNeptune2	LifeAct	<8	-	732	47.5	-	-	-	[4]
mNeptune2.5	LifeAct	<8	<8	732	47.5	-	-	-	[4]
mCardinal	LifeAct	>8	-	732	47.5	-	-	-	[4]
mRuby	LifeAct	<7	<7	732	9.5	-	-	-	[4]
mRuby2	LifeAct	<7	<7	732	9.5	-	-	-	[4]

Supplementary Table 3. List of primers used in this study.

Purpose	Primer	Sequence (5’-3’)
Monomerization of RpBphP2-derived iRFPs	Forward	GGCCGCCAAGCCTGCAAGAGGGTCGCCGAGAGG CTGGCCACTCAGATCGGCGTGATGGAAGAG
	Reverse	CTCTTCCATCACGCCGATCTGAGTGGCCAGCCTC TCGGCGACCCTCTTGCAGGCTTGGCGGCC
Monomerization of RpBphP6-derived iRFPs	Forward	CAGCGCGTCGCGGCCAAGAGGTTCGCCGAGAGG TTATCGACTCACTTCACCGCCGCCCAC
	Reverse	GTGGGCGGCGGTGAAGTGAGTCGATAACCTCTCG GCGAACCTCTTGGCCGCGACGCGCTG
Removing 12 aa from N-terminus of RpBphP1-derived miRFPs	Forward	CCACCGGTCGCCACCATGACCGCCTCTCATTCGA ATTGCG
	Reverse	CGCAATTCGAATGAGAGGCGGTCATGGTGGCGA CCGGTGG
Removing 13 aa from N-terminus of RpBphP1-derived miRFPs	Forward	CCACCGGTCGCCACCATGGCCTCTCATTCGAATT GCGAACATG
	Reverse	CATGTTCGCAATTCGAATGAGAGGCCATGGTGGC GACCGGTGG
Removing 14 aa from N-terminus of RpBphP1-derived miRFPs	Forward	GATCCACCGGTCGCCACCATGTCTCATTCGAATT GCGAACATGAAGAG
	Reverse	CTCTTCATGTTCGCAATTCGAATGAGACATGGTG GCGACCGGTGGATC
Removing 15 aa from N-terminus of RpBphP1-derived miRFPs	Forward	CCACCGGTCGCCACCATGCATTCGAATTGCGAAC ATGAAGAGATC

	Reverse	GATCTCTTCATGTTCGCAATTCGAATGCATGGTGGCGACCGGTGG
Removing 16 aa from N-terminus of RpBphP1-derived miRFPs	Forward	CCACCGGTCGCCACCATGTCGAATTGCGAACATGAAGAGATCC
	Reverse	GGATCTCTTCATGTTCGCAATTCGACATGGTGGCGACCGGTGG
Designing emiRFPs and C-terminal fusions of emiRFPs	Forward	GAACCGTCAGATCCGCTAGCCACCATGGCGGAAGGATCCGTCGCCAGGCAGCCTGACCTCTTGACCTGCGAACATGAAGAGATCC
	Reverse	GGATCTCTTCATGTTCGCAGGTCAAGAGGTCAGGCTGCCTGGCGACGGATCCTTCCGCCATGGTGGCTAGCGGATCTGACGGTTC
Designing N-terminal fusions of emiRFPs	Forward	ATCCACCGGTCGCCACCGCGGCCTCTCATTCGAATTGCG
	Reverse	GAGTGCGGCCGCTTAGCTCTCAAGCGCGGTG

```
BrBphP        1     --MPVPLTTPAFGHATLANCEREQIHLAGSIQPHGILLAVKEPDNVVIQASINAAEFLNT   58
mIFP          1     --MSVPLTTSAFGHAFLANCEREQIHLAGSIQPHGILLAVKEPDNVVIQASINAAEFLNT   58

RpBphP1       1     MVAGHASGSPAFGTADLSNCEREEIHLAGSIQPHGALLVVSEPDHRIIQASANAAEFLNL   60
miRFP670      1     MVAGHASGSPAFGTASHSNCEHEEIHLAGSIQPHGALLVVSEHDHRVIQASANAAEFLNL   60
emiRFP670     1     -----MAEGSVARQPDLLTCEHEEIHLAGSIQPHGALLVVSEHDHRVIQASANAAEFLNL   55
miRFP703      1     MVAGHASGSPAFGTASHSNCEHEEIHLAGSIQPHGALLVVSEHDHRVIQASANAAEFLNL   60
emiRFP703     1     -----MAEGSVARQPDLLTCEHEEIHLAGSIQPHGALLVVSEHDHRVIQASANAAEFLNL   55

RpBphP2       1     -----MTEGSVARQPDLSTCDDEPIHIPGAIQPHGLLLALAADMTIV-AGSDNLPELTGL   54
iRFP713/V256C 1     -----MAEGSVARQPDLLTCDDEPIHIPGAIQPHGLLLALAADMTIV-AGSDNLPELTGL   54
miRFP680      1     -----MAEGSVARQPDLLTCDDEPIHIPGAIQPHGLLLALAADMTIV-AGSDNLPELTGL   54
iRFP682       1     -----MAEGSVARQPDLLTCDDEPIHIPGAIQPHGLLLALAADMTIV-AGSDNLPELTGL   54
iRFP713       1     -----MAEGSVARQPDLLTCDDEPIHIPGAIQPHGLLLALAADMTIV-AGSDNLPELTGL   54
miRFP713      1     -----MAEGSVARQPDLLTCDDEPIHIPGAIQPHGLLLALAADMTIV-AGSDNLPELTGL   54
iRFP720       1     -----MAEGSVARQPDLLTCDDEPIHIPGAIQPHGLLLALAADMTIV-AGSDNLPELTGL   54
miRFP720      1     -----MAEGSVARQPDLLTCDDEPIHIPGAIQPHGLLLALAADMTIV-AGSDNLPELTGL   54

RpBphP6       1     ----------MPRKVDLTSCDREPIHIPGSIQPCGCLLACDAQAVRITRISENAGAFFGR   50
iRFP670       1     ----------MARKVDLTSCDREPIHIPGSIQPCGCLLACDAQAVRITRITENAGAFFGR   50
miRFP670-2    1     ----------MARKVDLTSCDREPIHIPGSIQPCGCLLACDAQAVRITRITENAGAFFGR   50
iRFP702       1     ----------MARKVDLTSCDREPIHIPGSIQPCGCLLACDAQAVRITRITENAGAFFGR   50
miRFP702      1     ----------MARKVDLTSCDREPIHIPGSIQPCGCLLACDAQAVRITRITENAGAFFGR   50
```

PAS

```
BrBphP        59    --NSVVGRPLRDLGGDLA---LQILPHLNGPLHLAPMTLRCTVGSPPRRVDCTVHRPSNG   113
mIFP          59    --NSVVGRPLRDLGGDLP---LQILPHLNGPLHLAPMTLRCTVGSPPRRVDCTIHRPSNG   113

RpBphP1       61    --GSVLGVPLAEIDGDLL---IKILPHLDPTAEGMPVAVRCRIGNPSTEYDGLMHRPPEG   115
miRFP670      61    --GSVLGVPLAEIDGDLL---IKILPHLDPTAEGMPVAVRCRIGNPSTEYCGLMHRPPEG   115
emiRFP670     56    --GSVLGVPLAEIDGDLL---IKILPHLDPTAEGMPVAVRCRIGNPSTEYCGLMHRPPEG   110
miRFP703      61    --GSVLGVPLAEIDGDLL---IKILPHLDPTAEGMPVAVRCRIGNPSTEYCGLMHRPPEG   115
emiRFP703     56    --GSVLGVPLAEIDGDLL---IKILPHLDPTAEGMPVAVRCRIGNPSTEYCGLMHRPPEG   110

RpBphP2       55    AIGALIGRSAADVFDSETHNRLTIALAEPGAAVGAPIAVGFTMR-KDAGFVGSWHRH-DQ   112
iRFP713/V256C 55    AIGALIGRSAADVFDSETHNRLTIALAEPGAAVGAPITVGFTMR-KDAGFIGSWHRH-DQ   112
miRFP680      55    AIGALIGRSAADVFDSETHNRLTIALAEPGAAVGAPITVGFTMR-KDAGFIGSWHRH-DQ   112
iRFP682       55    AIGALIGRSAADVFDSETHNRLTIALAEPGAAVGAPITVGFTMR-KDAGFIGSWHRH-DQ   112
iRFP713       55    AIGALIGRSAADVFDSETHNRLTIALAEPGAAVGAPITVGFTMR-KDAGFIGSWHRH-DQ   112
miRFP713      55    AIGALIGRSAADVFDSETHNRLTIALAEPGAAVGAPITVGFTMR-KDAGFIGSWHRH-DQ   112
iRFP720       55    AIGALIGRSAADVFDSETHNRLTIALAEPGAAVGAPITVGFTMR-KDAGFIGSWHRH-DQ   112
miRFP720      55    AIGALIGRSAADVFDSETHNRLTIALAEPGAAVGAPITVGFTMR-KDAGFIGSWHRH-DQ   112

RpBphP6       51    ET-PRVGELLADYFGETEAHALRNALAQSSDPKRPALIFGWRDGLTGRTFDISLHRH-DG   108
iRFP670       51    ET-PRVGELLADYFGETEAHALRNALAQSSDPKRPALIFGWRDGLTGRTFDISLHRH-DG   108
miRFP670-2    51    ET-PRVGELLADYFGETEAHALRNALAQSSDPKRPALIFGWRDGLTGRTFDISLHRH-DG   108
iRFP702       51    ET-PRVGELLADYFGETEAHALRNALAQSSDPKRPALIFGWRDGLTGRTFDISLHRH-DG   108
miRFP702      51    ET-PRVGELLADYFGETEAHALRNALAQSSDPKRPALIFGWRDGLTGRTFDISLHRH-DG   108
```

PAS

```
BrBphP        114   GLIVELEPATKTTNVAPA----LDGAFHRITSSSSLIGLCDETATIFREITGYDRVMVYR   169
mIFP          114   GLIVELEPATKTTNIAPA----LDGAFHRITSSSSLMGLCDETATIIREITGYDRVMVVR   169

RpBphP1       116   GLIIELERAGPPIDLSGT----LAPALERIRTAGSLRALCDDTALLFQQCTGYDRVMVYR   171
miRFP670      116   GLIIELERAGPSIDLSGT----LAPALERIRTAGSLRALCDDTVLLFQQCTGYDRVMVYR   171
emiRFP670     111   GLIIELERAGPSIDLSGT----LAPALERIRTAGSLRALCDDTVLLFQQCTGYDRVMVYR   166
miRFP703      116   GLIIELERAGPSIDLSGT----LAPALERIRTAGSLRALCDDTVLLFQQCTGYDRVMVYR   171
emiRFP703     111   GLIIELERAGPSIDLSGT----LAPALERIRTAGSLRALCDDTVLLFQQCTGYDRVMVYR   166
```

```
RpBphP2       113   LVFLELEPPQRDVAEPQAFFRRTNSAIRRLQAAETLESACAAAAQEVREITGFDRVMIYR   172
iRFP713/V256C 113   LIFLELEPPQRDVAEPQAFFRRTNSAIRRLQAAETLESACAAAAQEVRKITGFDRVMIYR   172
miRFP680      113   LIFLELEPPQRDVAEPQAFFRRTNSAIRRLQAAETLESACAAAAQEVRKITGFDRVMIYR   172
iRFP682       113   LIFLELEPPQRDVAEPQAFFRRTNSAIRRLQAAETLESACAAAAQEVRKITGFDRVMIYR   172
iRFP713       113   LIFLELEPPQRDVAEPQAFFRRTNSAIRRLQAAETLESACAAAAQEVRKITGFDRVMIYR   172
miRFP713      113   LIFLELEPPQRDVAEPQAFFRRTNSAIRRLQAAETLESACAAAAQEVRKITGFDRVMIYR   172
iRFP720       113   LIFLELEPPQRDVAEPQAFFRRTNSAIRRLQAAETLESACAAAAQEVRKITGFDRVMIYR   172
miRFP720      113   LIFLELEPPQRDVAEPQAFFRRTNSAIRRLQAAETLESACAAAAQEVRKITGFDRVMIYR   172

RpBphP6       109   TSIVEFEPAAADQADNPL--RLTRQIIARTKELKSLEEMAARVPRYLQAMLGYHRVMMYR   166
iRFP670       109   TSIIEFEPAAAEQADNPL--RLTRQIIARTKELKSLEEMAARVPRYLQAMLGYHRVMLYR   166
miRFP670-2    109   TSIIEFEPAAAEQADNPL--RLTRQIIARTKELKSLEEMAARVPRYLQAMLGYHRVMLYR   166
iRFP702       109   TSIVEFEPAAAEQADNPL--RLTRQIIARTKELKSLEEMAARVPRYLQAMLGYHRVMLYR   166
miRFP702      109   TSIIEFEPAAAEQADNPL--RLTRQIIARTKELKSLEEMAARVPRYLQAMLGYHRVMLYR   166
```

PAS GAF

```
BrBphP        170   FDEEGHGEVLSERRRPDLEAFLGNRYPASDIPQIARRLYERNRVRLLVDVNYTPVPLQPR   229
mIFP          170   FDEEGNGEILSERRRADLEAFLGNRYPASTIPQIARRLYEHNRVRLLVDVNYTPVPLQPR   229

RpBphP1       172   FDEQGHGEVFSERHVPGLESYFGNRYPSSDIPQMARRLYERQRVRVLVDVSYQPVPLEPR   231
miRFP670      172   FDEQGHGLVFSECHVPGLESYFGNRYPSSTVPQMARQLYVRQRVRVLVDVTYQPVPLEPR   231
emiRFP670     167   FDEQGHGLVFSECHVPGLESYFGNRYPSSTVPQMARQLYVRQRVRVLVDVTYQPVPLEPR   226
miRFP703      172   FDEQGHGLVFSECHVPGLESYFGNRYPSSLVPQMARQLYVRQRVRVLVDVTYQPVPLEPR   231
emiRFP703     167   FDEQGHGLVFSECHVPGLESYFGNRYPSSLVPQMARQLYVRQRVRVLVDVTYQPVPLEPR   226

RpBphP2       173   FASDFSGEVIAEDRCAEVESYLGLHFPASDIPAQARRLYTINPVRIIPDINYRPVPVTPD   232
iRFP713/V256C 173   FASDFSGEVIAEDRCAEVESKLGLHYPASTVPAQARRLYTINPVRIIPDINYRPVPVTPD   232
miRFP680      173   FASDFSGEVIAEDRCAEVESKLGLHYPASTVPAQARRLYTINPVRIIPDINYRPVPVTPD   232
iRFP682       173   FASDFSGVVIAEDRCAEVESKLGLHYPASAVPAQARRLYTINPVRIIPDINYRPVPVTPD   232
iRFP713       173   FASDFSGEVIAEDRCAEVESKLGLHYPASTVPAQARRLYTINPVRIIPDINYRPVPVTPD   232
miRFP713      173   FASDFSGEVIAEDRCAEVESKLGLHYPASTVPAQARRLYTINPVRIIPDINYRPVPVTPD   232
iRFP720       173   FASDFSGSVIAEDRCAEVESKLGLHYPASFIPAQARRLYTINPVRIIPDINYRPVPVTPD   232
miRFP720      173   FASDFSGSVIAEDRCAEVESKLGLHYPASFIPAQARRLYTINPVRIIPDINYRPVPVTPD   232

RpBphP6       167   FADDGSGKVIGEAKRSDLESFLGQHFPASDIPQQARLLYLKNAIRVISDSRGISSRIVPE   226
iRFP670       167   FADDGSGMVIGEAKRSDLESFLGQHFPASLVPQQARLLYLKNAIRVVSDSRGISSRIVPE   226
miRFP670-2    167   FADDGSGMVIGEAKRSDLESFLGQHFPASLVPQQARLLYLKNAIRVVSDSRGISSRIVPE   226
iRFP702       167   FADDGSGKVIGEAKRSDLESFLGQHFPASLVPQQARLLYLKNAIRVVSDSRGISSRIVPE   226
miRFP702      167   FADDGSGMVIGEAKRSDLESFLGQHFPASLVPQQARLLYLKNAIRVVSDSRGISSRIVPE   226
```

GAF

```
BrBphP        230   ISPLNGRDLDMSLSCLRSMSPIHQKYLQNMGVGATLVCSLMVSGRLWGLIACHHYEPRFV   289
mIFP          230   ISPLNGRDLDMSLSCLRSMSPIHQKYMQDMGVGATLVCSLMVSGRLWGLIACHHYEPRFV   289

RpBphP1       232   LSPLTGRDLDMSGCFLRSMSPIHLQYLKNMGVRATLVVSLVVGGKLWGLVACHHYLPRFI   291
miRFP670      232   LSPLTGRDLDMSGCFLRSMSPCHLQFLKDMGVRATLAVSLVVGGKLWGLVVCHHYLPRFI   291
emiRFP670     227   LSPLTGRDLDMSGCFLRSMSPCHLQFLKDMGVRATLAVSLVVGGKLWGLVVCHHYLPRFI   286
miRFP703      232   LSPLTGRDLDMSGCFLRSMSPIHLQFLKDMGVRATLAVSLVVGGKLWGLVVCHHYLPRFI   291
emiRFP703     227   LSPLTGRDLDMSGCFLRSMSPIHLQFLKDMGVRATLAVSLVVGGKLWGLVVCHHYLPRFI   286

RpBphP2       233   LNPVTGRPIDLSFAILRSVSPVHLEYMRNIGMHGTMSISILRGERLWGLIACHHRKPNYV   292
iRFP713/V256C 233   LNPVTGRPIDLSFAILRSVSPCHLEFMRNIGMHGTMSISILRGERLWGLIVCHHRTPYYV   292
miRFP680      233   LNPVTGRPIDLSFAILRSVSPCHLEFMRNIGMHGTMSISILRGERLWGLIVCHHRTPYYV   292
iRFP682       233   LNPVTGRPIDLSFAILRSVSPCHLEFMRNIGMHGTMSISILRGERLWGLIVCHHRTPYYV   292
iRFP713       233   LNPVTGRPIDLSFAILRSVSPVHLEFMRNIGMHGTMSISILRGERLWGLIVCHHRTPYYV   292
miRFP713      233   LNPVTGRPIDLSFAILRSVSPVHLEFMRNIGMHGTMSISILRGERLWGLIVCHHRTPYYV   292
iRFP720       233   LNPVTGRPIDLSFAILRSVSPNHLEFMRNIGMHGTMSISILRGERLWGLIVCHHRTPYYV   292
miRFP720      233   LNPVTGRPIDLSFAILRSVSPNHLEFMRNIGMHGTMSISILRGERLWGLIVCHHRTPYYV   292
```

```
RpBphP6        227   RDAS-GAALDLSFAHLRSVSPIHLEYLRNMGVSASMSLSIIIDGTLWGLIACHHYEPRAV   285
iRFP670        227   HDAS-GAALDLSFAHLRSISPCHLEFLRNMGVSASMSLSIIIDGTLWGLIICHHYEPRAV   285
miRFP670-2     227   HDAS-GAALDLSFAHLRSISPCHLEFLRNMGVSASMSLSIIIDGTLWGLIICHHYEPRAV   285
iRFP702        227   HDAS-GAALDLSFAHLRSISPIHLEFLRNMGVSASMSLSIIIDGTLWGLIICHHYEPRAV   285
miRFP702       227   HDAS-GAALDLSFAHLRSISPCHLEFLRNMGVSASMSLSIIIDGTLWGLIICHHYEPRAV   285
                     GAF

BrBphP         290   PFDIRAAGEALAETCAIRIAALESFAQSQSE   320
mIFP           290   PFHIRAAGEALAETCAIRIATLESFAQSQSK   320

RpBphP1        292   HFELRAICELLAEAIATRITALES-------   315
miRFP670       292   RFELRAICKRLAERIATRITALES-------   315
emiRFP670      287   RFELRAICKRLAERIATRITALES-------   310
miRFP703       292   RFELRAICKRLAERIATRITALES-------   315
emiRFP703      287   RFELRAICKRLAERIATRITALES-------   310

RpBphP2        293   DLDGRQACELVAQVLAWQIGVMEE-------   316
iRFP713/V256C  293   DLDGRQACELVAQVLAWQIGVMEE-------   316
miRFP680       293   DLDGRQACKRVAERLATQIGVMEE-------   316
iRFP682        293   DLDGRQACELVAQVLAWQIGVMEE-------   316
iRFP713        293   DLDGRQACELVAQVLAWQIGVMEE-------   316
miRFP713       293   DLDGRQACKRVAERLATQIGVMEE-------   316
iRFP720        293   DLDGRQACELVAQVLAWQIGVMEE-------   316
miRFP720       293   DLDGRQACKRVAERLATQIGVMEE-------   316

RpBphP6        286   PMAQRVAAEMFADFFSLHFTAAHHQR-----   311
iRFP670        286   PMAQRVAAEMFADFLSLHFTAAHHQR-----   311
miRFP670-2     286   PMAQRVAAKRFAERLSTHFTAAHHQR-----   311
iRFP702        286   PMAQRVAAEMFADFLSLHFTAAHHQR-----   311
miRFP702       286   PMAQRVAAKRFAERLSTHFTAAHHQR-----   311
                     GAF
```

Supplementary Figure 1. Alignment of the amino acid sequences of reported NIR FPs with parental BrBphP, RpBphP1, RpBphP2 and RpBphP6 bacterial phytochromes. The residues substituted in the monomerized proteins based on alignment with RpBphP1-derived miRFPs are in yellow, the residues substituted in emiRFPs based on alignment with RpBphP2-derived miRFPs are marked green. BV-binding Cys residues are marked grey.

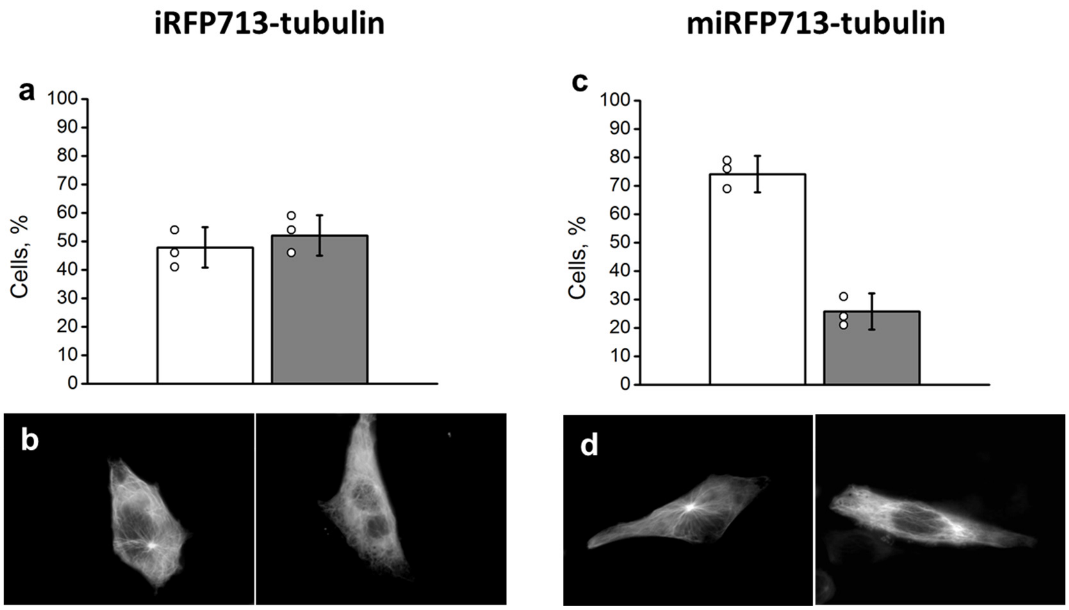

Supplementary Figure 2. Comparison of localization efficiency for tubulin fusions of dimeric and monomeric iRFP713 variants. (**a, c**) Percent of HeLa cells with proper localization (white column) or aberrant localization (grey column) of fluorescently labeled tubulin. (**b, d**) Widefield images showing representative cells with proper (left) or aberrant (right) localization of the construct. Error bards are double s.e.m, N=3 transfection experiments with at least 50 cells total for each fusion construct.

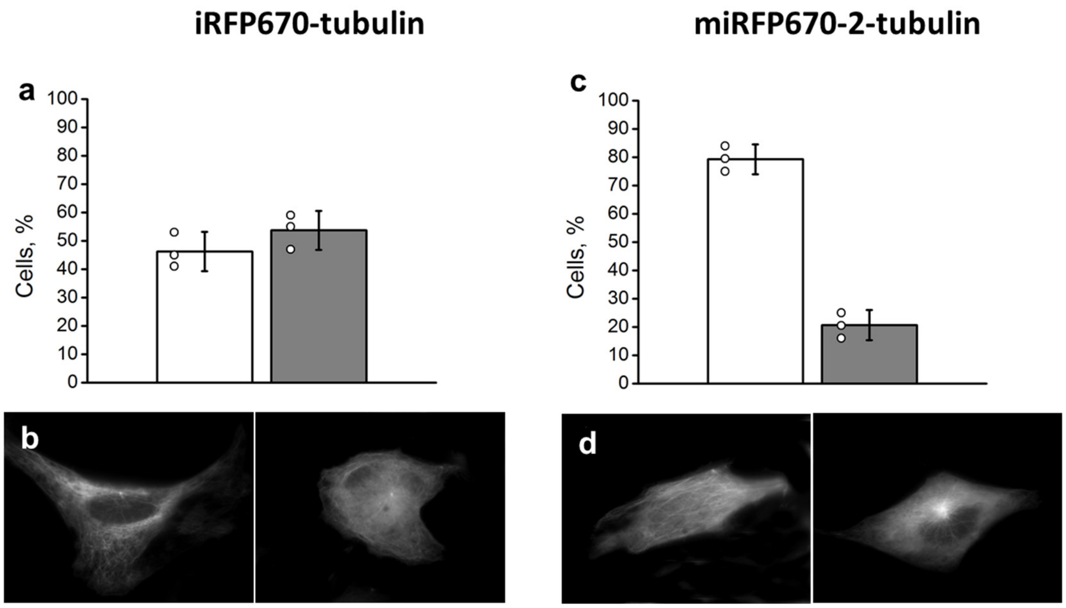

Supplementary Figure 3. Comparison of localization efficiency for tubulin fusions of dimeric and monomeric iRFP670 variants. (**a, c**) Percent of HeLa cells with proper localization (white column) or aberrant localization (grey column) of fluorescently labeled tubulin. (**b, d**) Widefield images showing representative cells with proper (left) or aberrant (right) localization of the construct. Error bards are double s.e.m, N=3 transfection experiments with at least 50 cells total for each fusion construct.

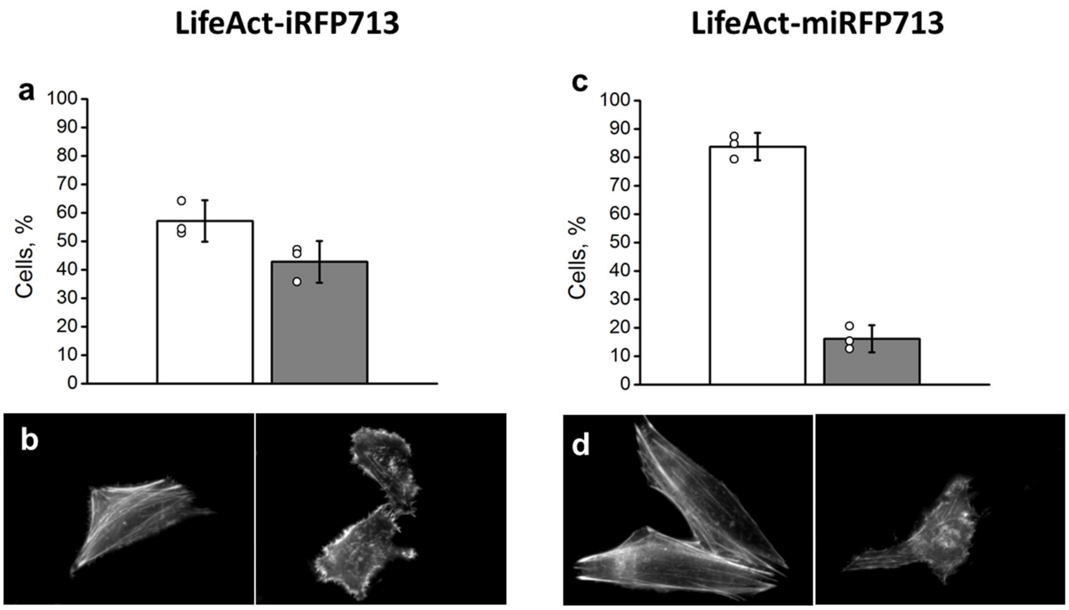

Supplementary Figure 4. Comparison of localization efficiency for LifeAct fusions of dimeric and monomeric iRFP713 variants. (**a, c**) Percent of HeLa cells with proper localization (white column) or aberrant localization (grey column) of fluorescently labeled tubulin. (**b, d**) Widefield images showing representative cells with proper (left) or aberrant (right) localization of the construct. Error bards are double s.e.m, N=3 transfection experiments with at least 50 cells total for each fusion construct.

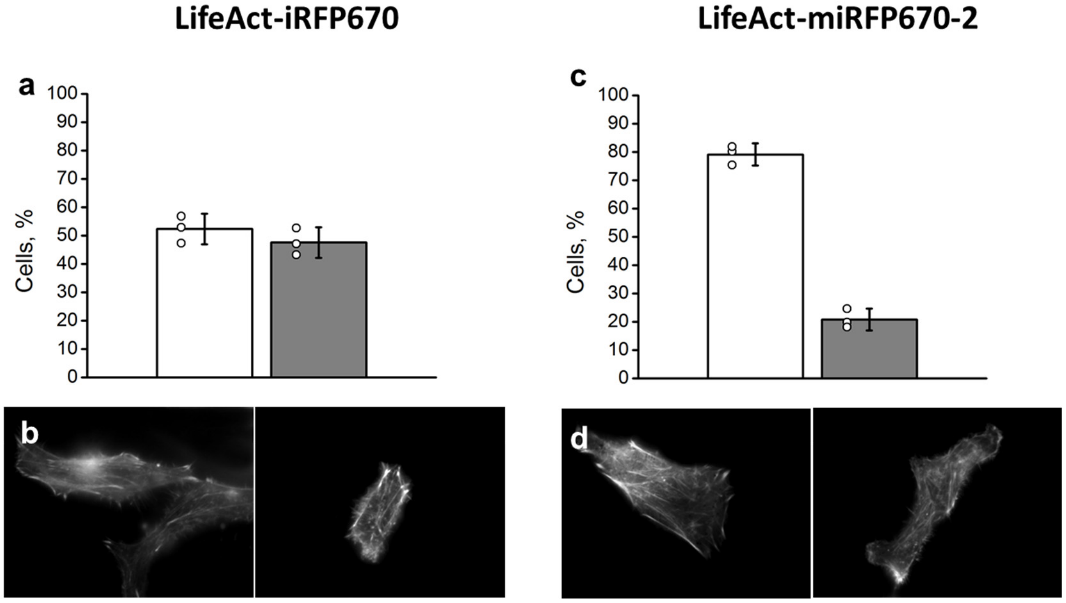

Supplementary Figure 5. Comparison of localization efficiency for LifeAct fusions of dimeric and monomeric iRFP670 variants. (**a, c**) Percent of HeLa cells with proper localization (white column) or aberrant localization (grey column) of fluorescently labeled tubulin. (**b, d**) Widefield images showing representative cells with proper (left) or aberrant (right) localization of the construct. Error bards are double s.e.m, N=3 transfection experiments with at least 50 cells total for each fusion construct.

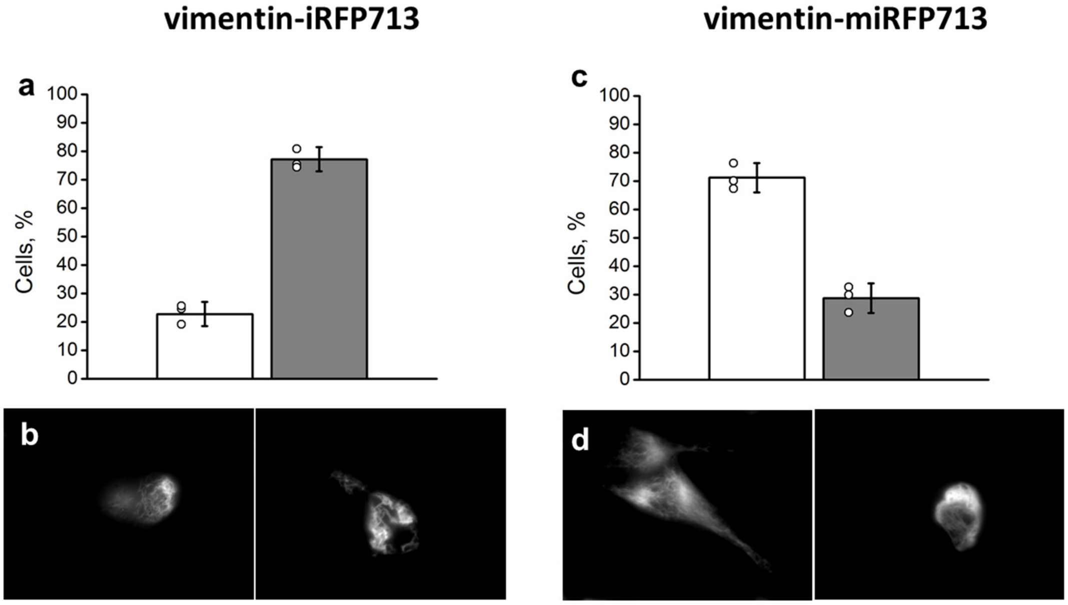

Supplementary Figure 6. Comparison of localization efficiency for vimentin fusions of dimeric and monomeric iRFP713 variants. (**a, c**) Percent of HeLa cells with proper localization (white column) or aberrant localization (grey column) of fluorescently labeled tubulin. (**b, d**) Widefield images showing representative cells with proper (left) or aberrant (right) localization of the construct. Error bards are double s.e.m, N=3 transfection experiments with at least 50 cells total for each fusion construct.

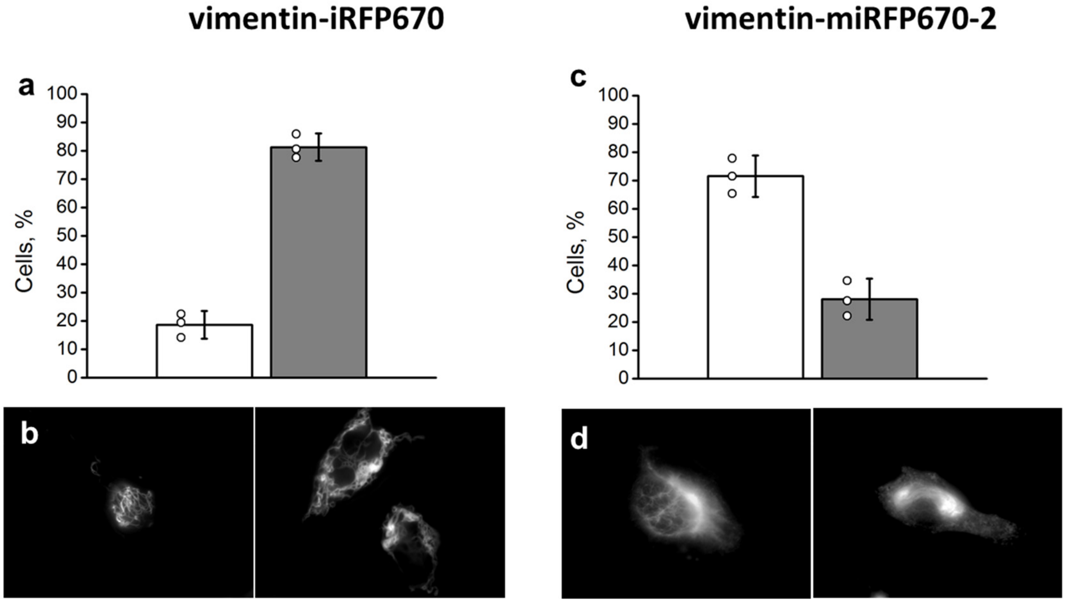

Supplementary Figure 7. Comparison of localization efficiency for vimentin fusions of dimeric and monomeric iRFP670 variants. (**a, c**) Percent of HeLa cells with proper localization (white column) or aberrant localization (grey column) of fluorescently labeled tubulin. (**b, d**) Widefield images showing representative cells with proper (left) or aberrant (right) localization of the construct. Error bards are double s.e.m, N=3 transfection experiments with at least 50 cells total for each fusion construct.

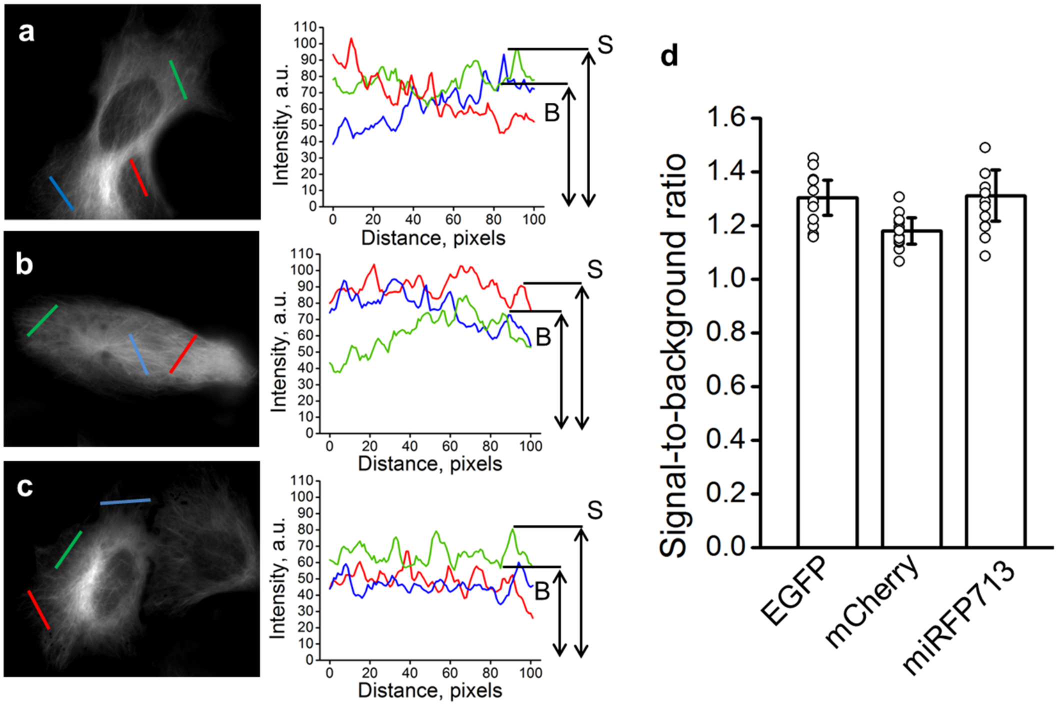

Supplementary Figure 8. Signal-to-background ratio for tubulin fusions of EGFP, mCherry and miRFP713. (**a**-**c**) Widefield images of HeLa cells expressing EGFP-tubulin (a), mCherry-tubulin (b) and miRFP713-tubulin (c). For each cell, 3 profiles were plotted across cell body. (**d**) Signal-to-background ratio was measured as a ratio of fluorescence intensity of microtubules to fluorescence intensity of surrounding cytoplasm (indicated in the profiles as S and B, respectively). Error bards are double s.e.m, N ≥ 15 cells for each fusion construct.

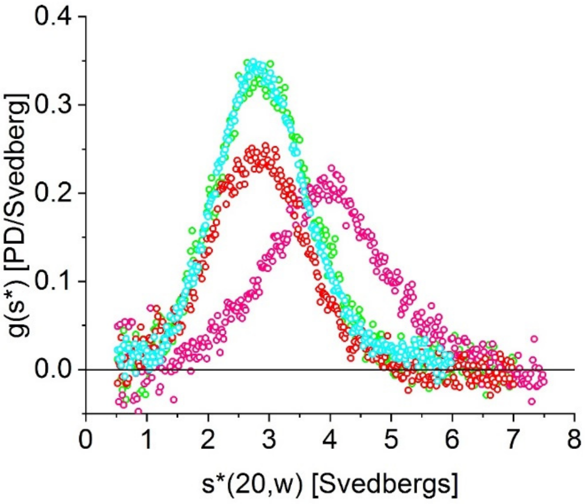

Supplementary Figure 9. Sedimentation analysis of monomerized iRFP variants. Sedimentation velocity analytical ultracentrifugation of monomerized miRFP713 (representative RpBphP2-derived FP, in red), monomerized miRFP670-2 (representative RpBphP6-derived FP, in green), control monomeric RpBphP1-derived miRFP670 (in cyan) and dimeric RpBphP2-derived iRFP720 (in magenta) run in the same conditions. The proteins were analyzed at concentrations of 15 µM in PBS buffer at 20ºC, the time-derivative method was used. Overlay of the sedimentation coefficient distributions are shown. Monomeric NIR FPs showed peaks with the maxima centered at a sedimentation coefficient of ~2.8-2.85 S that corresponds to the monomer (MW=35±3kDa). Control dimeric iRFP720 showed the peak at ~4.0 S that corresponds to the dimer.

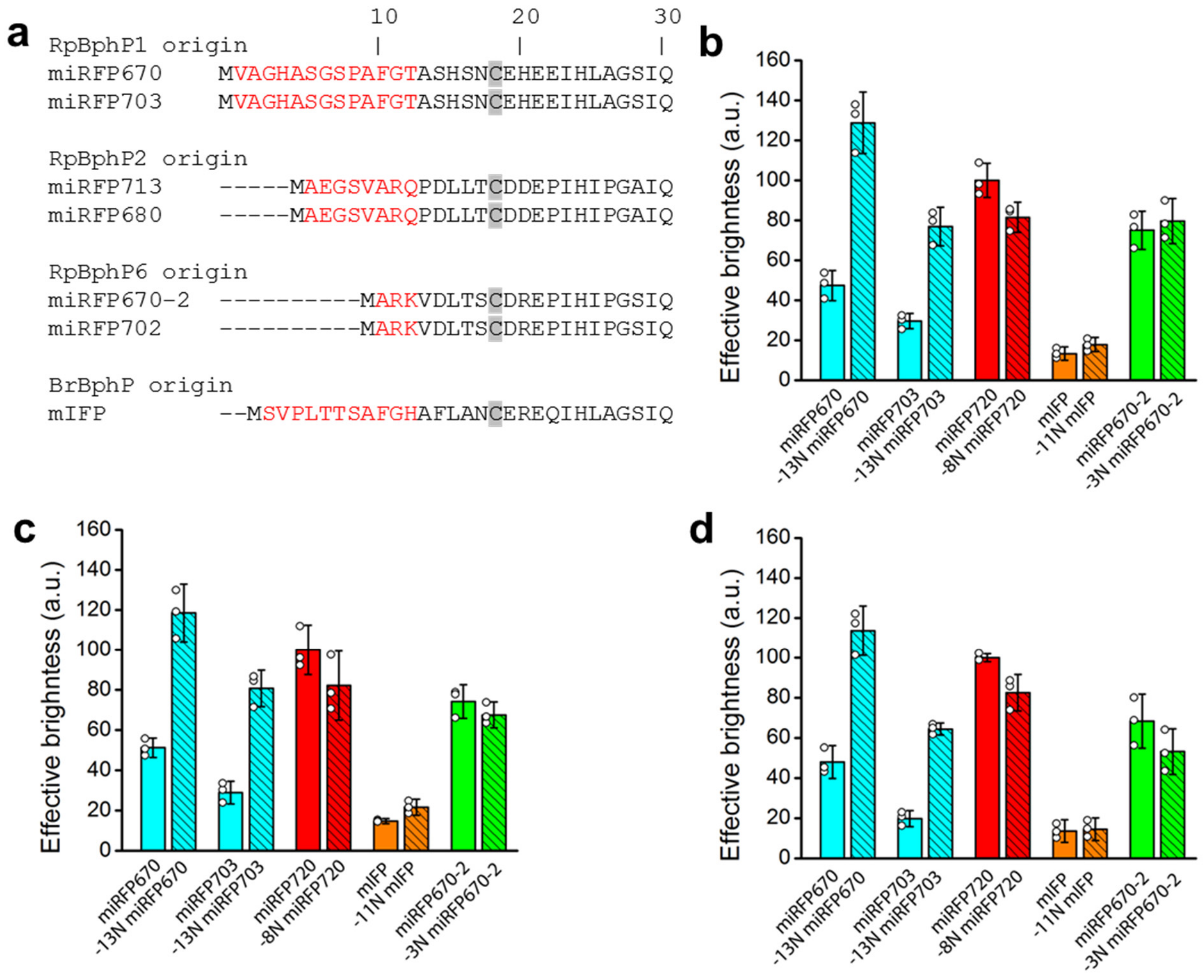

Supplementary Figure 10. Effect of N-terminus truncation on brightness of NIR mFPs derived from different BphPs in mammalian cells. (**a**) Alignment of N-termini of various BphP precursors of monomeric NIR FPs. (**b**) Effective brightness of miRFP variants with the original (plane) and shortened N-terminus (diagonal pattern) in live COS-1 cells 72 h after transfection. The effective brightness of miRFP720 is assumed to be 100%. (**c,d**) Same as in (a) but in live HeLa (c) and live NIH3T3 (d) cells. Error bards are double s.e.m, N=3 transfection experiments.

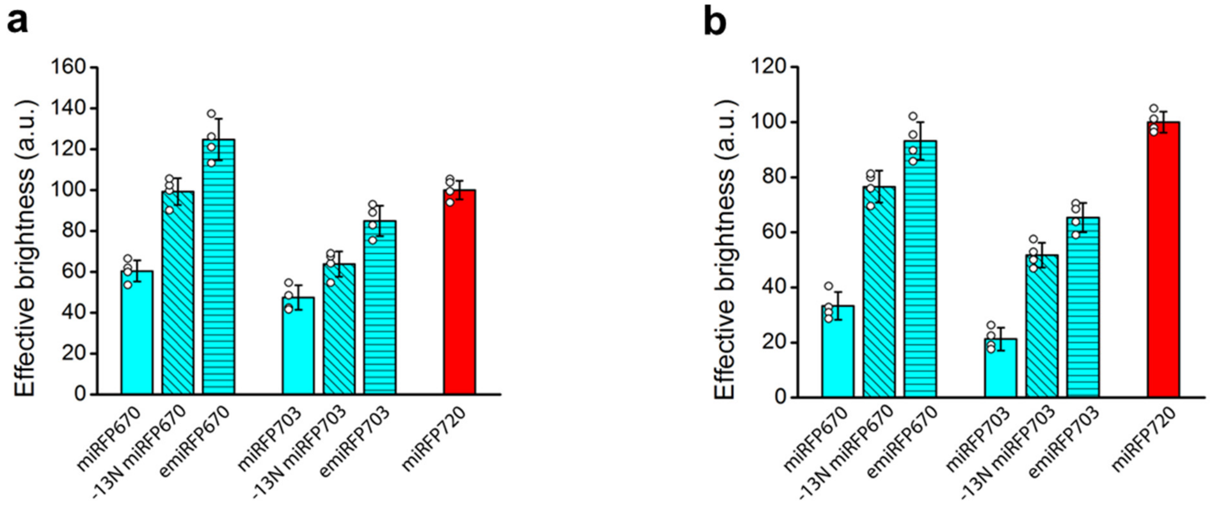

Supplementary Figure 11. Effect of N-terminus on brightness of RpBphP1-derived NIR mFPs in mammalian cells. (**a,b**) Effective brightness of miRFP670 and miRFP703, their variants with 13 amino acid residues removed from N-terminus, and their variants with N-termini from RpBphP2. Measurements were performed 72 h after transfection in live HeLa cells (a) and live NIH3T3 cells (b). Effective brightness of miRFP720 was assumed to be 100%. Error bards are double s.e.m, N=3 transfection experiments.

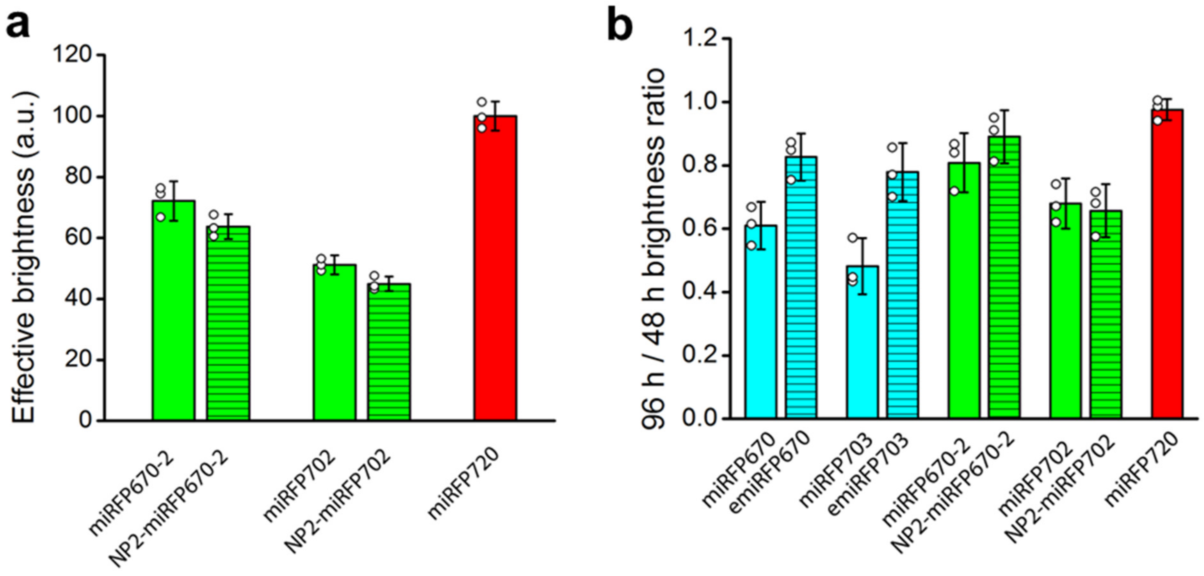

Supplementary Figure 12. The effect of N-terminus in miRFPs on the effective brightness and protein stability. (**a**) Effective brightness of miRFPs derived from RpBphP6 with truncated N-terminus and their variants with N-termini of RpBphP2. (**b**) Ratio of effective brightness of miRFPs and emiRFPs derived from RpBphP1 and miRFPs derived from RpBphP6 at 48 h and 96 h. Error bars, s.e.m. (n=3; transfection experiments). Error bards are double s.e.m, N=3 transfection experiments.

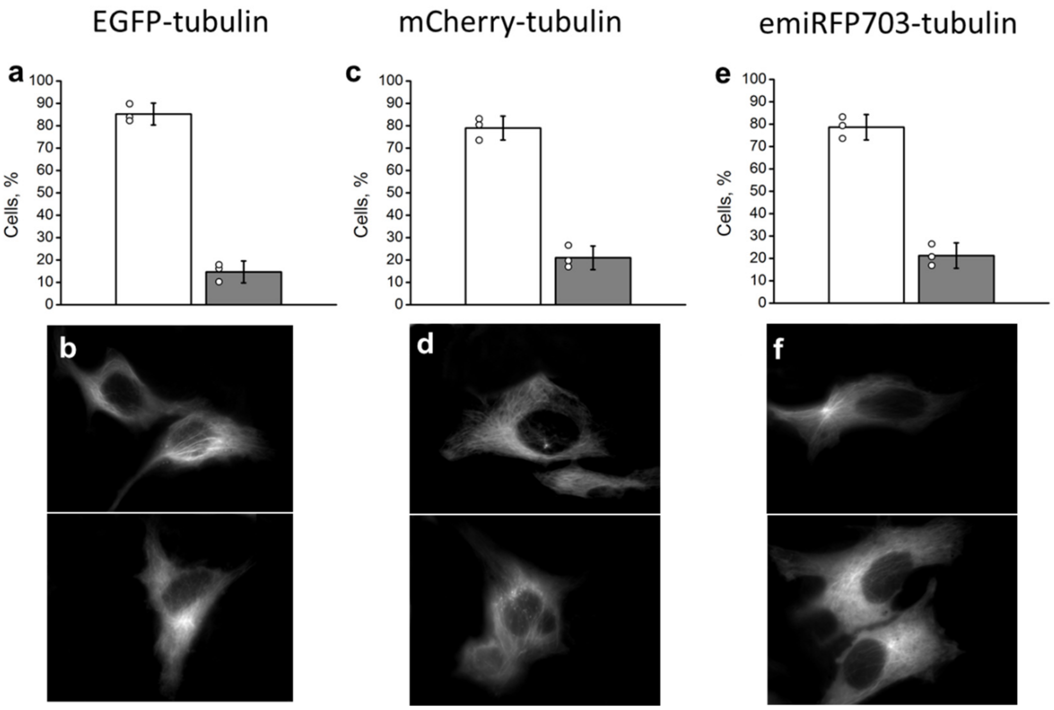

Supplementary Figure 13. Comparison of localization efficiency for tubulin fusions of EGFP, mCherry and emiRFP703. (**a, c, e**) Percent of HeLa cells with proper localization (white column) or aberrant localization (grey column) of fluorescently labeled tubulin. (**b, d, f**) Widefield images showing representative cells with proper (top) or aberrant (bottom) localization of the construct. Error bards are double s.e.m, N=3 transfection experiments with at least 50 cells total for each fusion construct.

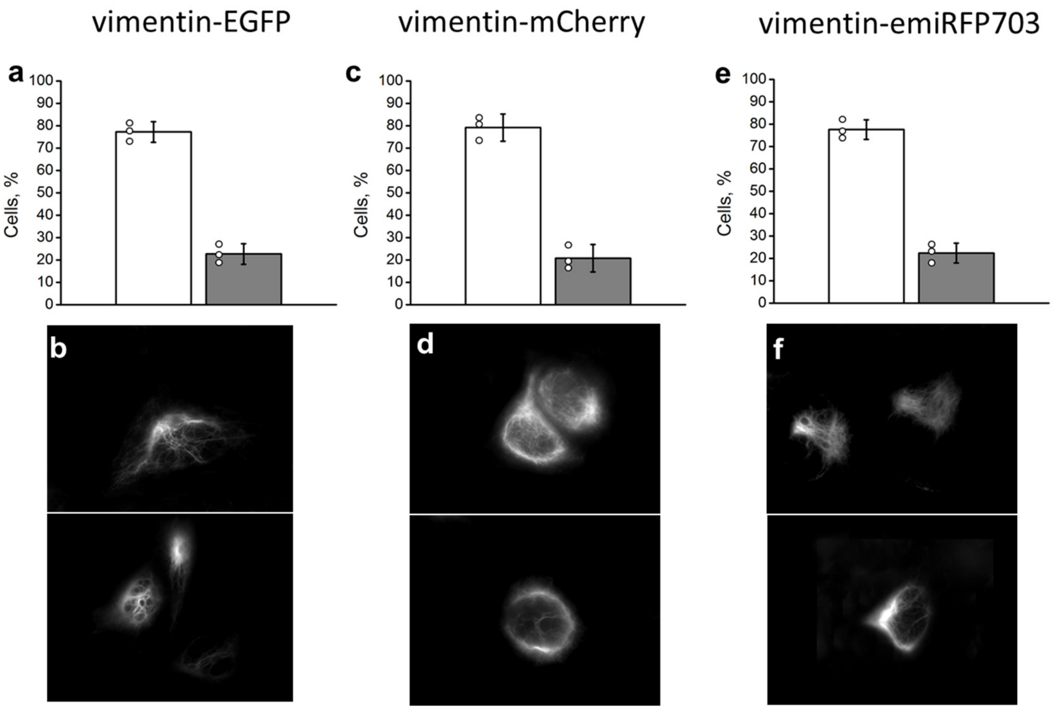

Supplementary Figure 14. Comparison of localization efficiency for vimentin fusions of EGFP, mCherry and emiRFP703. (**a, c, e**) Percent of HeLa cells with proper localization (white column) or aberrant localization (grey column) of fluorescently labeled tubulin. (**b, d, f**) Widefield images showing representative cells with proper (top) or aberrant (bottom) localization of the construct. Error bards are double s.e.m, N=3 transfection experiments with at least 50 cells total for each fusion construct.

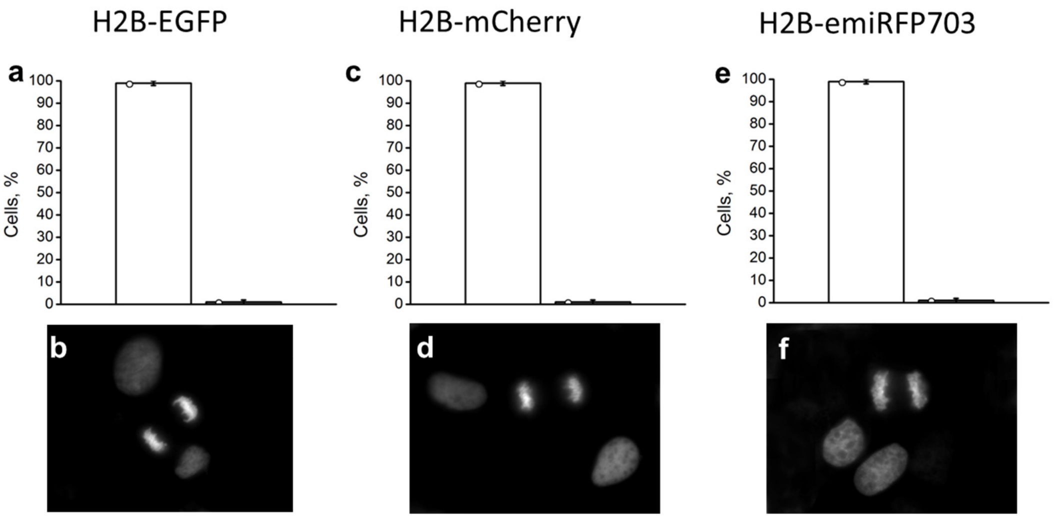

Supplementary Figure 15. Comparison of localization efficiency for H2B fusions of EGFP, mCherry and emiRFP703. (**a, c, e**) Percent of HeLa cells with proper localization (white column) or aberrant localization (grey column) of fluorescently labeled tubulin. (**b, d, f**) Widefield images showing representative cells expressing H2B-EGFP (b), H2B-mCherry (d) and H2B-emiRFP703 (f) constructs. Error bards are double s.e.m, N=3 transfection experiments with at least 50 cells total for each fusion construct.

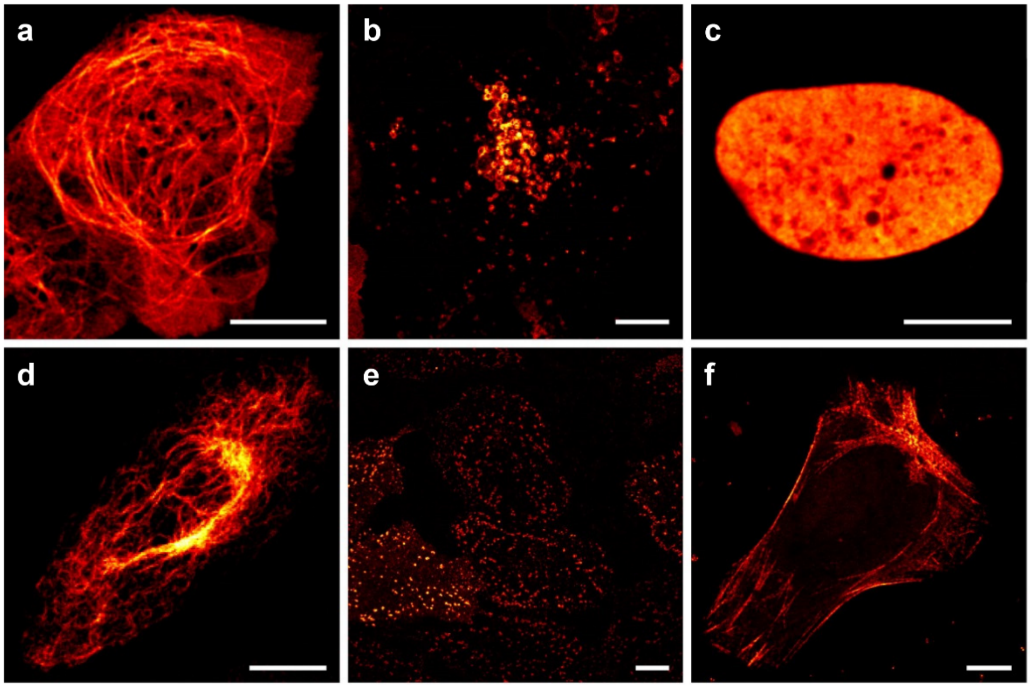

Supplementary Figure 16. Confocal imaging of HeLa and U2OS cells expressing emiRFP-variants. Confocal images of cells expressing (**a**) emiRFP703-tubulin, (**b**) LAMP1-emiRFP670, (**c**) H2B-emiRFP670, (**d**) vimentin-emiRFP703, (**e**) emiRFP703-clathrin, and (**f**) emiRFP703-myosin. The cells were transfected for 24-72 h. Scale bars, 10 μm.

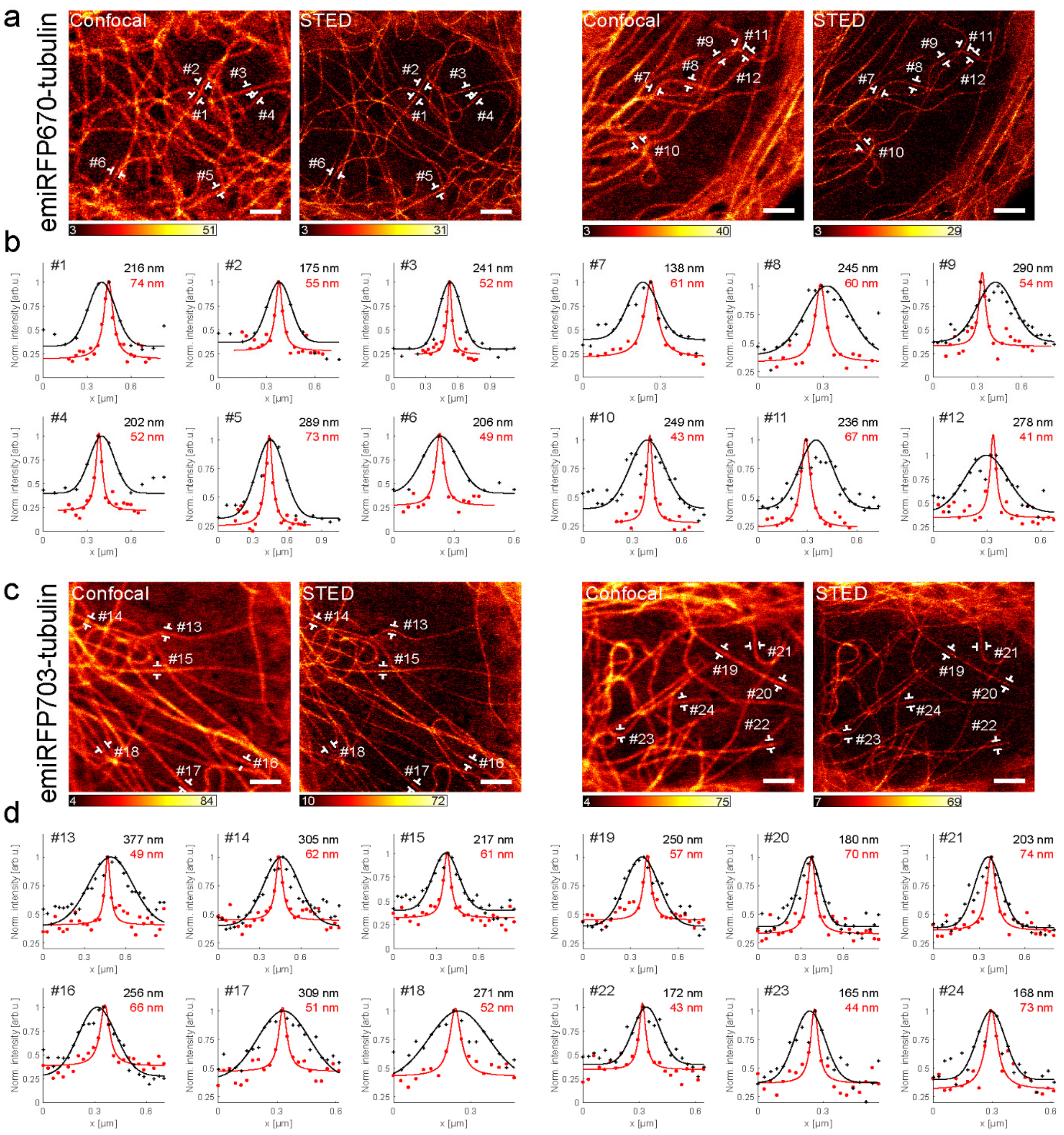

Supplementary Figure 17. STED imaging of HeLa cells with emiRFP670-tubulin and emiRFP703-tubulin. (**a**) Confocal and STED images of HeLa cells transfected with emiRFP670-tubulin. (**b**) #1-#12 shows line profiles and corresponding fits from the marked lines in the images in panel a. The fits are Gaussian fits to the confocal data (black solid lines) and Lorentzian fits to the STED data (red solid lines). (**c**) Confocal and STED images of HeLa cells transfected with emiRFP703-tubulin. (**d**) #13-#24 shows line profiles and corresponding fits from the marked lines in the images in a. The fits are Gaussian fits to the confocal data (black solid lines) and Lorentzian fits to the STED data (red solid lines). Line profiles are averaged over a width of 3 pixels. Scale bars, 2 µm.

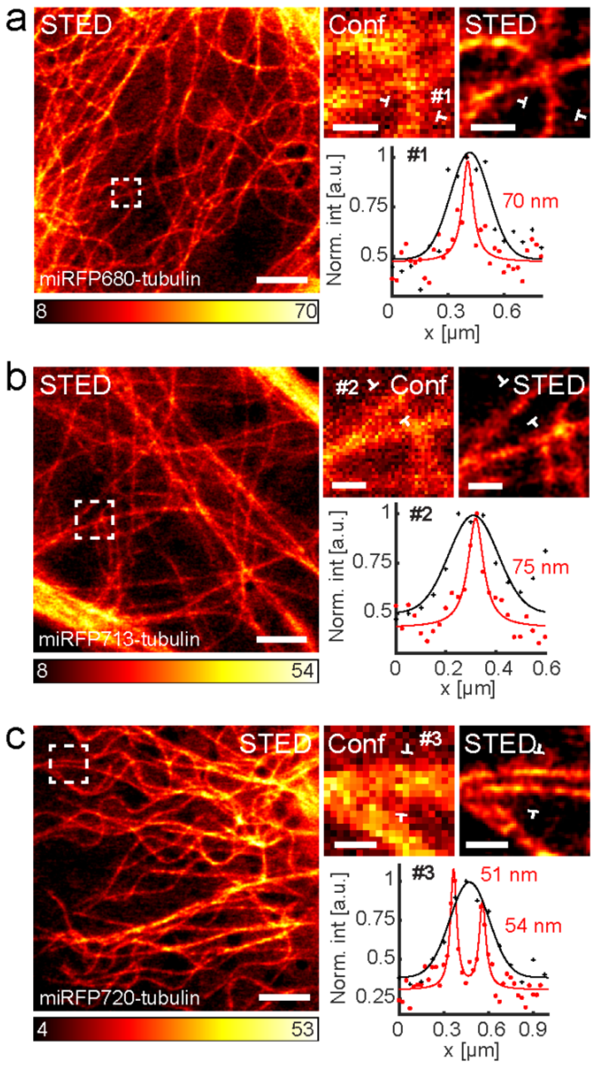

Supplementary Figure 18. STED imaging of U2OS cells expressing tubulin fused to miRFP680, miRFP713 and miRFP720. (**a**) Confocal and STED images of a U2OS cell expressing miRFP680-tubulin. #1 shows a line profile from the zoom-in of the region of interest, with a Gaussian fit for the confocal data (black solid line) and a Lorentzian fit for the STED data (red solid line). (**b**) Confocal and STED images of a U2OS cell expressing miRFP713-tubulin. #2 shows a line profile from the zoom-in of the region of interest, with a Gaussian fit for the confocal data (black solid line) and a Lorentzian fit for the STED data (red solid line). (**c**) Confocal and STED images of a U2OS cell expressing miRFP720-tubulin. #3 shows a line profile from the zoom-in of the region of interest, showing two tubules in close proximity, only resolvable in the STED image. The line profile is fitted with a Gaussian function for the confocal data (black solid line) and a double Lorentzian function for the STED data (red solid line).

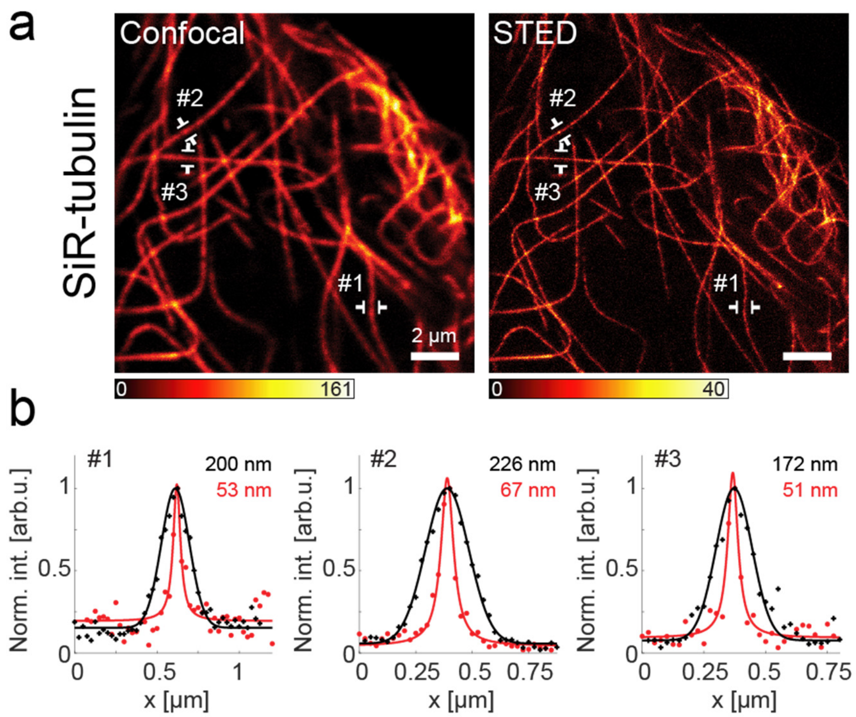

Supplementary Figure 19. STED imaging of U2OS cells with SiR-tubulin. (**a**) Confocal and STED image of U2OS cells with SiR-tubulin. (**b**) #1-#3 shows line profiles and corresponding fits from the marked lines in the images in panel a. The fits are Gaussian fits to the confocal data (black solid lines) and Lorentzian fits to the STED data (red solid lines). Line profiles are averaged over a width of 3 pixels. Scale bars, 2 µm.

Supplementary Note 1. Comparison of the developed NIR FPs with SiR dye.

To evaluate the competitiveness of the new engineered set of enhanced miRFPs with established far-red fluorescent proteins, such as SNIFP and mGarnet2, and tubulin-labeling dyes, such as SiR-tubulin, we compare the photostability, STED illumination dose, signal-to-noise (SNR) and signal-to-background (SBR) ratios of these FPs and dyes (Supplementary Tables 1 and 2).

Comparing the photostability of the probes in terms of possible recorded frames, for optimized STED imaging, the emiRFP703 performs well in the comparison with the previously published far-red FPs (Supplementary Table 2). In our photostability measurements (Fig. 5) we get 33 STED frames before the integrated fluorescence signal drops to 1/e of the initial value. We see that the emiRFP703 can deliver three times as many frames as compared with measurements with the comparable FPs expressed together with vimentin, namely SNIFP and mGarnet2. Additionally, the STED illumination dose on the sample is at least 11x and up to 36x lower for emiRFP703 as compared to the other far-red FPs, something that is always important to consider in live-cell measurements. This low value is thanks to the engineered properties of the new emiRFP proteins, whose emission spectra more efficiently overlaps with the STED wavelength of 775 nm, without causing additional direct STED beam excitation or re-excitation.

When it comes to the SNR the emiRFP703 and emiRFP670 variants expressed with tubulin, as an example of the emiRFP-family, compares well when compared to imaging with the well-established SiR-tubulin dye (Supplementary Fig. 18 and Supplementary Table 1). The images from which the SNR- and SBR-values are calculated are optimized for image quality in terms of for example resolution, and we get the same resolution with the emiRFPs (Fig. 4, Supplementary Fig. 17) as with SiR-tubulin (Supplementary Fig. 19). Moreover, the total STED illumination dose used in the example of emiRFP703 is two-times lower than that used when imaging SiR-tubulin. We see that the SBR is lower for emiRFP670-tubulin and emiRFP703-tubulin than for SiR-tubulin. This owes to the fact that the emiRFPs are expressed with all α-tubulin, inevitably also the cytosolic portion, while SiR-tubulin on the other hand only labels the polymerized α-tubulin in the microtubules by using the drug Docetaxel [5, 6]. This creates an almost background-free image in the case of SiR-tubulin, while the emiRFP703-tubulin image will have a cytosolic background ever present. Indeed, looking at emiRFP703 expressed with a protein that does not show noteworthy cytosolic background levels such as vimentin (Fig. 4d) we get a similar SNR-value and indeed also SBR-value as for SiR-tubulin, with as much as a 16x lower STED illumination dose.

Supplementary References

1. Kamper, M., Ta, H., Jensen, N.A., Hell, S.W. & Jakobs, S. Near-infrared STED nanoscopy with an engineered bacterial phytochrome. *Nat Commun* **9**, 4762 (2018).
2. Hense, A. et al. Monomeric Garnet, a far-red fluorescent protein for live-cell STED imaging. *Sci Rep* **5**, 18006 (2015).
3. Matela, G. et al. A far-red emitting fluorescent marker protein, mGarnet2, for microscopy and STED nanoscopy. *Chem Commun (Camb)* **53**, 979-982 (2017).
4. Wegner, W. et al. In vivo mouse and live cell STED microscopy of neuronal actin plasticity using far-red emitting fluorescent proteins. *Sci Rep* **7**, 11781 (2017).
5. Lukinavicius, G. et al. Fluorogenic probes for live-cell imaging of the cytoskeleton. *Nat Methods* **11**, 731-733 (2014).
6. Lukinavicius, G. et al. Fluorescent dyes and probes for super-resolution microscopy of microtubules and tracheoles in living cells and tissues. *Chem Sci* **9**, 3324-3334 (2018).
