# Supplementary material for: A set of monomeric near-infrared fluorescent proteins for multicolor imaging across scales
Source: Nat Commun. 2020 Jan 13;11:239. doi: 10.1038/s41467-019-13897-6 (PMC6957686; doi:10.1038/s41467-019-13897-6)
Supplement: Supplementary file 2 — Reporting Summary [file 41467_2019_13897_MOESM2_ESM.pdf]

nature research

Corresponding author(s): Vladislav Verkhusha

Last updated by author(s): Nov 26, 2019

Reporting Summary

Nature Research wishes to improve the reproducibility of the work that we publish. This form provides structure for consistency and transparency in reporting. For further information on Nature Research policies, see Authors & Referees and the Editorial Policy Checklist .

Statistics

For all statistical analyses, confirm that the following items are present in the figure legend, table legend, main text, or Methods section.

n/a	Confirmed	
☐	☒	The exact sample size (*n*) for each experimental group/condition, given as a discrete number and unit of measurement
☐	☒	A statement on whether measurements were taken from distinct samples or whether the same sample was measured repeatedly
☐	☒	The statistical test(s) used AND whether they are one- or two-sided<br>*Only common tests should be described solely by name; describe more complex techniques in the Methods section.*
☒	☐	A description of all covariates tested
☒	☐	A description of any assumptions or corrections, such as tests of normality and adjustment for multiple comparisons
☒	☐	A full description of the statistical parameters including central tendency (e.g. means) or other basic estimates (e.g. regression coefficient) AND variation (e.g. standard deviation) or associated estimates of uncertainty (e.g. confidence intervals)
☒	☐	For null hypothesis testing, the test statistic (e.g. *F*, *t*, *r*) with confidence intervals, effect sizes, degrees of freedom and *P* value noted<br>*Give P values as exact values whenever suitable.*
☒	☐	For Bayesian analysis, information on the choice of priors and Markov chain Monte Carlo settings
☒	☐	For hierarchical and complex designs, identification of the appropriate level for tests and full reporting of outcomes
☒	☐	Estimates of effect sizes (e.g. Cohen's *d*, Pearson's *r*), indicating how they were calculated

Our web collection on statistics for biologists contains articles on many of the points above.

Software and code

Policy information about availability of computer code

Data collection	Ultracentrifugation scans were analyzed with DCDTþ v.2.4.2 software (J.S. Philo, 2006). The fluorescent microscope was operated with a SlideBook v.6.0.8 software (Intelligent Imaging Innovations). Flow cytometry data were collected using BD FACSDiva v.8.0.1 (BD Biosciences) software and mean fluorescence intensity was calculated with FlowJo v.7.6.2 software (FlowJo LLC). STED imaging was performed on a custom-built STED setup. STED image acquisition and hardware control is done through the Imspector software (Max-Planck Innovation) and the custom-written Python-based open-source microscope control software Tempesta (https://github.com/TestaLab/Tempesta, https://github.com/jonatanalvelid/Tempesta-RedSTED). Animal imaging data were analyzed using Living Image 3.0 software (Perkin Elmer/Caliper Life Sciences).
Data analysis	Data fitting and statistical analysis were performed using an OriginPro v.9.2.196 software (OriginLab). Line profile fitting with Lorentzian functions for STED images and Gaussian functions for confocal images, both including a background term, was done using the Curve fitting toolbox in MATLAB (MathWorks). Line profiles were always taken and analysis was always performed on the raw data. Where noted, presented STED images have been deconvolved with a Richardson-Lucy algorithm in Imspector.

For manuscripts utilizing custom algorithms or software that are central to the research but not yet described in published literature, software must be made available to editors/reviewers. We strongly encourage code deposition in a community repository (e.g. GitHub). See the Nature Research guidelines for submitting code & software for further information.

Data

Policy information about availability of data

All manuscripts must include a data availability statement. This statement should provide the following information, where applicable:

- Accession codes, unique identifiers, or web links for publicly available datasets
- A list of figures that have associated raw data
- A description of any restrictions on data availability

The main data supporting the findings of this study are available within the Article and its Supplementary information. The additional data are available from the corresponding author on reasonable request. The emiRFP670, miRFP680, emiRFP703 and miRFP713 nucleotide sequences will be available on GenBank before the

final submission of the manuscript.

Field-specific reporting

Please select the one below that is the best fit for your research. If you are not sure, read the appropriate sections before making your selection.

☒ Life sciences ☐ Behavioural & social sciences ☐ Ecological, evolutionary & environmental sciences

For a reference copy of the document with all sections, see nature.com/documents/nr-reporting-summary-flat.pdf

Life sciences study design

All studies must disclose on these points even when the disclosure is negative.

Sample size	No sample-size calculations were performed. Standard N>3 independent experiments were performed for most cases, unless noted in the figure legend. For STED imaging sample sizes were chosen in order to check that we recorded images with similar quality and resolution independently from different cells, different days of transfection and different days of recording.
Data exclusions	No data were excluded.
Replication	STED and confocal imaging performance for all presented protein variants and labeled structures was tested and reproduced in different cells, different days of transfection and different days of recording. All other attempts at replication were successful.
Randomization	Not applicable
Blinding	Not applicable

Reporting for specific materials, systems and methods

We require information from authors about some types of materials, experimental systems and methods used in many studies. Here, indicate whether each material, system or method listed is relevant to your study. If you are not sure if a list item applies to your research, read the appropriate section before selecting a response.

Materials & experimental systems

n/a	Involved in the study
☒	☐ Antibodies
☐	☒ Eukaryotic cell lines
☒	☐ Palaeontology
☐	☒ Animals and other organisms
☒	☐ Human research participants
☒	☐ Clinical data

Methods

n/a	Involved in the study
☒	☐ ChIP-seq
☐	☒ Flow cytometry
☒	☐ MRI-based neuroimaging

Eukaryotic cell lines

Policy information about cell lines

Cell line source(s)	HeLa (CCL-2), HEK293 (CRL-1573), NIH3T3 (CRL-1658), COS-1 (CRL-1650) and U2OS (HTB-96) cell lines were obtained from ATCC
Authentication	Cell lines were not additionally authenticated.
Mycoplasma contamination	Cell lines were not additionally tested for mycoplasma.
Commonly misidentified lines (See ICLAC register)	No commonly misidentified cell lines were used.

Animals and other organisms

Policy information about studies involving animals; ARRIVE guidelines recommended for reporting animal research

Laboratory animals	The Swiss Webster 2- to 3-month-old female mice (National Cancer Institute, NIH) with body weights of 22–25 g were used.
Wild animals	No wild animals were used in the study.
Field-collected samples	No field-collected samples were used in the study.

Ethics oversight	All animal experiments were performed in an AAALAC-approved facility using protocols approved by the Albert Einstein College of Medicine Animal Usage Committee.

Note that full information on the approval of the study protocol must also be provided in the manuscript.

Flow Cytometry

Plots

Confirm that:

- [x] The axis labels state the marker and fluorochrome used (e.g. CD4-FITC).
- [x] The axis scales are clearly visible. Include numbers along axes only for bottom left plot of group (a 'group' is an analysis of identical markers).
- [ ] All plots are contour plots with outliers or pseudocolor plots.
- [x] A numerical value for number of cells or percentage (with statistics) is provided.

Methodology

Sample preparation	Prior to acquisition, cell pellets were washed with PBS and diluted in cold PBS to density 500,000 cells per ml . At least 30,000 cells per sample were recorded.
Instrument	BD LSRII flow cytometer.
Software	BD FACSDiva v.8.0.1 (BD Biosciences), FlowJo v.7.6.2 software (FlowJo LLC).
Cell population abundance	Not applicable
Gating strategy	Initial gates - FSC-A/SSC-A to discriminate cells from debris; then cells were gated in FSC-W/FSC-A to discriminate single cells; then cells were gated in SSC-W/SSC-A to discriminate live cells. Resulted population were analyzed on SSC-A/APC and SSC-A/Alexa700 plots to find cells expressing miRFPs. Mock transected cells were used for selecting negative population.

- [ ] Tick this box to confirm that a figure exemplifying the gating strategy is provided in the Supplementary Information.
